# *ProteoSeeker*: A Feature‐Rich Metagenomic Analysis Tool for Accessible and Comprehensive Metagenomic Exploration

**DOI:** 10.1002/advs.202414877

**Published:** 2025-03-25

**Authors:** Georgios Filis, Dimitra Bezantakou, Konstantinos Rigkos, Despina Noti, Pavlos Saridis, Dimitra Zarafeta, Georgios Skretas

**Affiliations:** ^1^ Institute for Bioinnovation Biomedical Sciences Research Center “Alexander Fleming” Vari 16672 Greece; ^2^ Institute of Chemical Biology National Hellenic Research Foundation Athens 11635 Greece; ^3^ Department of Informatics and Telecommunications National and Kapodistrian University of Athens Athens 16122 Greece; ^4^ Department of Biological Applications and Technologies University of Ioannina Ioannina 45500 Greece; ^5^ Faculty of Biology National and Kapodistrian University of Athens Athens 15772 Greece

**Keywords:** bioinformatics pipeline, command‐line tool, metagenomic analysis, protein discovery, shotgun sequencing, taxonomic analysis, whole‐genome sequencing

## Abstract

The vast majority of microbial diversity remains unculturable, limiting access to novel biotechnological resources. Advances in metagenomics have expanded the understanding of microbial communities, yet targeted protein discovery remains challenging. This study introduces *ProteoSeeker*, a command‐line tool for streamlined metagenomic protein identification and annotation. *ProteoSeeker* operates in two primary modes: i) Seek mode, which screens the proteins according to user‐defined protein families, and ii) Taxonomy mode, which uncovers the taxonomy of the host organisms. By automating key steps, *ProteoSeeker* reduces computational complexity, enabling time‐efficient and comprehensive metagenomic analysis for both specialized and nonspecialized users. The efficiency of *ProteoSeeker* to achieve targeted enzyme discovery is demonstrated by identifying extremophilic enzymes with desired biochemical features, such as amylases for starch hydrolysis and carbonic anhydrases for CO₂ capture applications. By democratizing functional metagenomics, *ProteoSeeker* is anticipated to accelerate biotechnology, synthetic biology, and biomedical research and innovation.

## Graphical abstract

*ProteoSeeker* is a new metagenomic analysis tool that accelerates protein discovery. By automating key steps, it reduces computational complexity and enables comprehensive dataset analysis, performing protein identification, screening based on user‐defined protein families, and taxonomic analysis. Available on GitHub and shipped as a Docker image, it operates with minimal input, allowing non‐experts to explore large‐scale metagenomic data.

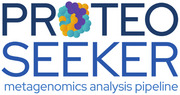

## 1 Introduction

A total of ≈80% of the microbial diversity on Earth remains unculturable, posing a significant limitation in unlocking the functional potential encoded within its genetic content.^[^ ^1^ ^]^ Metagenomic analysis can bypass this bottleneck by providing access to the collective genomic information contained in environmental and other sources.^[^ ^2^ ^]^ The exponential growth of publicly available (meta)genomic data, driven by advances in DNA sequencing technologies, underscores the critical need for computational tools to process this vast information reservoir and identify genes encoding biotechnology‐relevant proteins, which can significantly drive research and innovation in various fields, such as biotechnology, synthetic biology, and biomedicine.^[^ ^3^ ^]^ Additionally, function‐driven metagenomics serves as a cornerstone for the identification of novel genes, leading to the discovery of proteins and enzymes with new and/or enhanced functional traits.^[^ ^4^ ^]^

A promising area of metagenomic analysis toward the discovery of new and potentially useful proteins involves studying extremophiles, i.e., microorganisms residing in extreme environmental conditions. Adapted to challenging environments with high or low temperatures, pH, or salinity, extremophiles can harbor unique biocatalysts, known as extremozymes. These enzymes have demonstrated value in diverse applications spanning agriculture, biochemistry, biomedicine, and beyond.^[^ ^5^ ^]^ Leveraging bioinformatic methods is essential for analyzing the vast and continuously increasing metagenomic data, thus enabling and accelerating the identification, annotation, and functional prediction of novel extremozymes.

While metagenomic analysis using bioinformatic algorithms is highly advantageous, it still faces certain technological constraints. One of the fundamental processes in metagenomics analysis is the generation of “metagenome‐assembled genomes” (MAGs), typically obtained from whole‐(meta)genome sequencing (W(M)GS). This method involves the fragmentation of the genetic material, enabling the thorough sequencing of all DNA fragments in a sample, even at a single‐strain level, which allows for detailed subsequent profiling.^[^ ^6^ ^]^

Analyzing metagenomic data presents significant challenges, particularly in selecting and combining tools to perform separate tasks, such as sequence assembly, binning, and annotation. These tasks are complex and time‐consuming, requiring a deep understanding of various computational methods and their compatibility. Over the past decade, several benchmarking studies have evaluated the performance of various metagenomic bioinformatic tools and workflows, providing valuable insights into accuracy and efficiency across different analytical steps.^[^ ^7^, ^8^, ^9^ ^]^ Well‐established platforms, such as MG‐RAST, IMG/M and MGnify, are widely recognized choices, offering broad functionality for tasks like sequence assembly, binning, and annotation, thereby greatly facilitating the early phases of metagenomic analysis.^[^ ^10^, ^11^, ^12^ ^]^ The main strength of these platforms is their ability to provide a broad taxonomic or functional overview of the analyzed metagenomes to the user. However, these tools have not been specifically designed to provide in‐depth, user‐configurable options needed for more specialized goals, such as targeted protein discovery. Consequently, and for such purposes, many researchers must piece together multiple external tools, each with their own parameters and outputs, leading to increased complexity and decreased comprehensibility and user‐friendliness. Thus, there still remains a critical unmet need for developing comprehensive integrated pipelines for automated analysis of (meta)genomic data to facilitate more specialized research goals, such as the discovery of new proteins with desired functional features.^[^ ^13^ ^]^

To address these challenges, we introduce *ProteoSeeker*, a command‐line tool specifically designed for comprehensive functional analysis of sequencing data acquired from environmental or other metagenomes and, by extension, of genomic and proteomic data. The main focus of *ProteoSeeker* is to identify proteins, screen them based on user‐defined protein families, and/or determine the taxonomy of their host organisms. Building on the capabilities of existing computational tools, *ProteoSeeker* unifies protein annotation, protein‐domain identification, protein‐family prediction, and taxonomic profiling within a single framework. Its command‐line interface is designed to minimize technical barriers, making comprehensive metagenomic data analysis accessible to both non‐specialized users with limited computational expertise and experienced bioinformaticians alike. Moreover, its modularity allows researchers to tailor specific steps to specific data types and research objectives, with minimal user input. We anticipate that *Proteoseeker's* comprehensive approach to functional profiling will advance the field toward more standardized, yet adaptable, metagenomic workflows.

The key advantage of *ProteoSeeker* lies in its ease of use and high degree of automation, distinguishing it from fragmented approaches that require stringing together multiple standalone tools. By offering an end‐to‐end workflow‐from raw sequencing reads to function and taxonomy‐based insights, *ProteoSeeker* ensures consistent parameter settings, reduces tool‐incompatibility issues, and shortens overall analysis time. Consequently, *ProteoSeeker* not only streamlines specialized discovery efforts, such as the identification of novel extremozymes but also promotes the broader adoption of metagenomic analysis by simplifying and accelerating critical bioinformatic processes.

By democratizing the exploration of metagenomic data, *ProteoSeeker* is poised to accelerate novel protein discovery and drive innovation and sustainability goals across diverse sectors, ranging from agriculture and pharmaceuticals to environmental remediation and beyond, within the rapidly evolving landscape of biotechnological research and innovation.

## 2 Results

### 2.1 Overview

*ProteoSeeker* is a novel, highly comprehensive pipeline that combines state‐of‐the‐art software for the analysis of whole‐(meta)genome sequencing (W(M)GS) data, contigs, genomes, and proteomic data. Metagenomic data may originate from environmental, clinical, or other samples. It is designed to run effectively on limited computational resources without hindering scalability. Comprehensibility is achieved through the automation of key processes, such as read preprocessing, contig assembly, gene prediction, putative protein screening, and taxonomic analysis. *ProteoSeeker* is designed to run with minimal user input. The user has to provide only an SRA code from the SRA database of NCBI^[^ ^14^, ^15^ ^]^ or a dataset and‐in certain cases‐at least one protein family code to initiate the analysis. The tools and their versions included in *ProteoSeeker* version 1.0.0 (“v.1.0.0”) are found in the Supporting Information (Supporting Text: 1.1) of this article.

The tool offers two main functional modes termed “seek” and “taxonomy”. In both modes, *ProteoSeeker* identifies proteins encoded by predicted protein‐coding regions in the contigs assembled from the sequencing reads or in the genomic data provided (e.g., contigs, genomes). In the seek mode, the identified putative proteins are filtered based on the protein family (or families) specified by the user, while in the taxonomy mode, the identified putative proteins undergo taxonomic classification. Users have the flexibility to select the application of either one or both modes in a single *ProteoSeeker* run.

**Figure 1:**
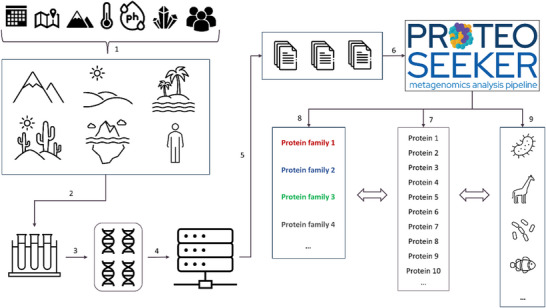
*ProteoSeeker's* application within the framework of metagenomic protein discovery. 1) The targeted sampling environments are selected by defining the specific conditions/origin of interest (organism, temperature, pH, salinity etc.) of the proteins to be discovered and 2) the metagenomic sample is collected from the selected environments. 3) The DNA of the metagenomic sample is isolated and prepared for sequencing. 4) The metagenomic material is sequenced using Next‐Generation Sequencing protocols. 5) Files containing reads are generated and may be uploaded to publicly available databases (as the SRA) alongside their metadata. 6) The *ProteoSeeker* user provides an SRA code or a dataset as input. 7) *ProteoSeeker* identifies putative proteins from the assembled reads. Users can select to apply the “seek” mode, the “taxonomy” mode or both. 8) In the seek mode certain identified proteins are associated with the user‐defined protein families based on their corresponding Pfam profiles and/or protein names. This mode is used to uncover novel proteins with targeted functionalities. 9) In its taxonomy mode *ProteoSeeker* performs binning and taxonomy classification of the identified proteins.

Each individual tool included in the *ProteoSeeker* pipeline has been specifically selected and optimized to facilitate rapid analysis of the input data. The protein annotation process aims to provide users with insights on which candidate proteins may be suitable for further examination, based on the provided protein annotation. The *ProteoSeeker* pipeline is designed to be highly versatile, enabling users to selectively omit analysis stages depending on the specific scope of their work. **Figure** **1** illustrates the role of *ProteoSeeker* in the general workflow of protein discovery through metagenomic analysis.

**Figure 2:**
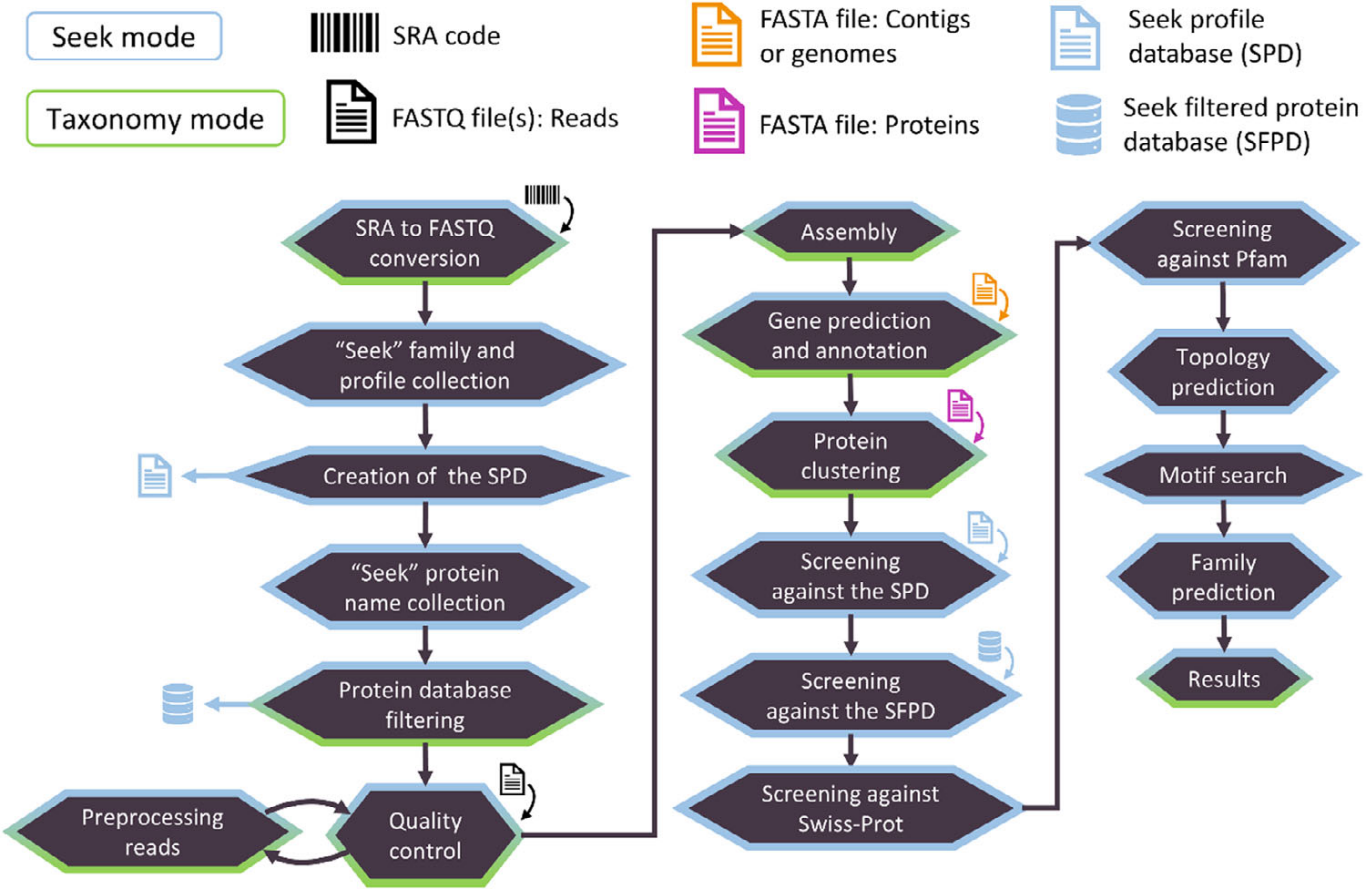
The stages of the “seek” mode of *ProteoSeeker*. *ProteoSeeker* offers the functionalities of the seek mode (blue) and of the “taxonomy” mode (green). Each stage is colored based on the mode it belongs to. The possible types of input for *ProteoSeeker* include an SRA code, reads in FASTQ files, and contigs or genomes or proteins in FASTA format. If an SRA code is provided the corresponding SRA and FASTQ files are generated. The “seek” protein families are selected based on the input “seek” family codes and their profiles are collected. The “seek profile database” (SPD) is created. The “seek” protein names of the selected families are collected, and the protein database is filtered based on these names creating the “seek filtered protein database” (SFPD). The reads of the FASTQ files undergo several quality checks by FastQC. The reads are preprocessed by BBDuk and then reanalyzed by FastQC. The preprocessed reads are assembled into contigs by Megahit. Protein coding regions (pcdrs) are predicted in the contigs by FragGeneScanRs. CD‐HIT is used to reduce the redundancy of the pcdrs. The pcdrs are screened against the SPD with HMMER. Any pcdr with at least one hit based on the latter screening is retained (protein set 1). The rest of the pcdrs are screened against the SFPD with DIAMOND and only those with a hit of low enough E‐value are retained (protein set 2). Protein set 1 is screened against the UniProtKB/Swiss‐Prot database with DIAMOND. Both protein sets are screened against all the profiles of the Pfam database with HMMER. Topology predictions are performed by Phobius. Motifs provided by the user are screened against each protein. The protein family of each protein is predicted. Annotation files are written.

*ProteoSeeker* offers a multitude of options regarding the user's desired output. Some of these options control the workflow of the pipeline. As mentioned above, either the seek or the taxonomy mode or both can be applied in a single *ProteoSeeker* run. The seek mode of *ProteoSeeker* includes three types of analysis (“type 1”, “type 2”, “type 3”). Type 1 analysis includes searching for proteins containing at least one domain corresponding to a profile associated with the selected protein families. Type 2 analysis includes searching for putative proteins with hits having E‐values lower than a specific score. These hits are acquired by running DIAMOND^[^ ^16^ ^]^ to screen the putative proteins against a filtered protein database. Type 3 analysis applies both type 1 and type 2 analyses in a single run. The filtering of the protein database has been applied based on the protein names associated with the selected protein families and the provided protein database. Type 1 analysis generates results comprising putative proteins, which are likely to belong to the selected protein families. Type 2 analysis is more likely to generate results that comprise candidate proteins, which significantly differ from the characterized family members of the selected protein families. These results might include proteins, which are part of the selected families, while sharing distant amino acid sequences with the other family members or belong to new protein families related to the selected ones. A summary of the results documented in the annotation files generated from a *ProteoSeeker* run after applying both the seek and taxonomy modes, can be found in Table S1 (Supporting Information). The stages of the pipeline in the seek mode of *ProteoSeeker* are described in **Figure** **2**.

**Figure 3:**
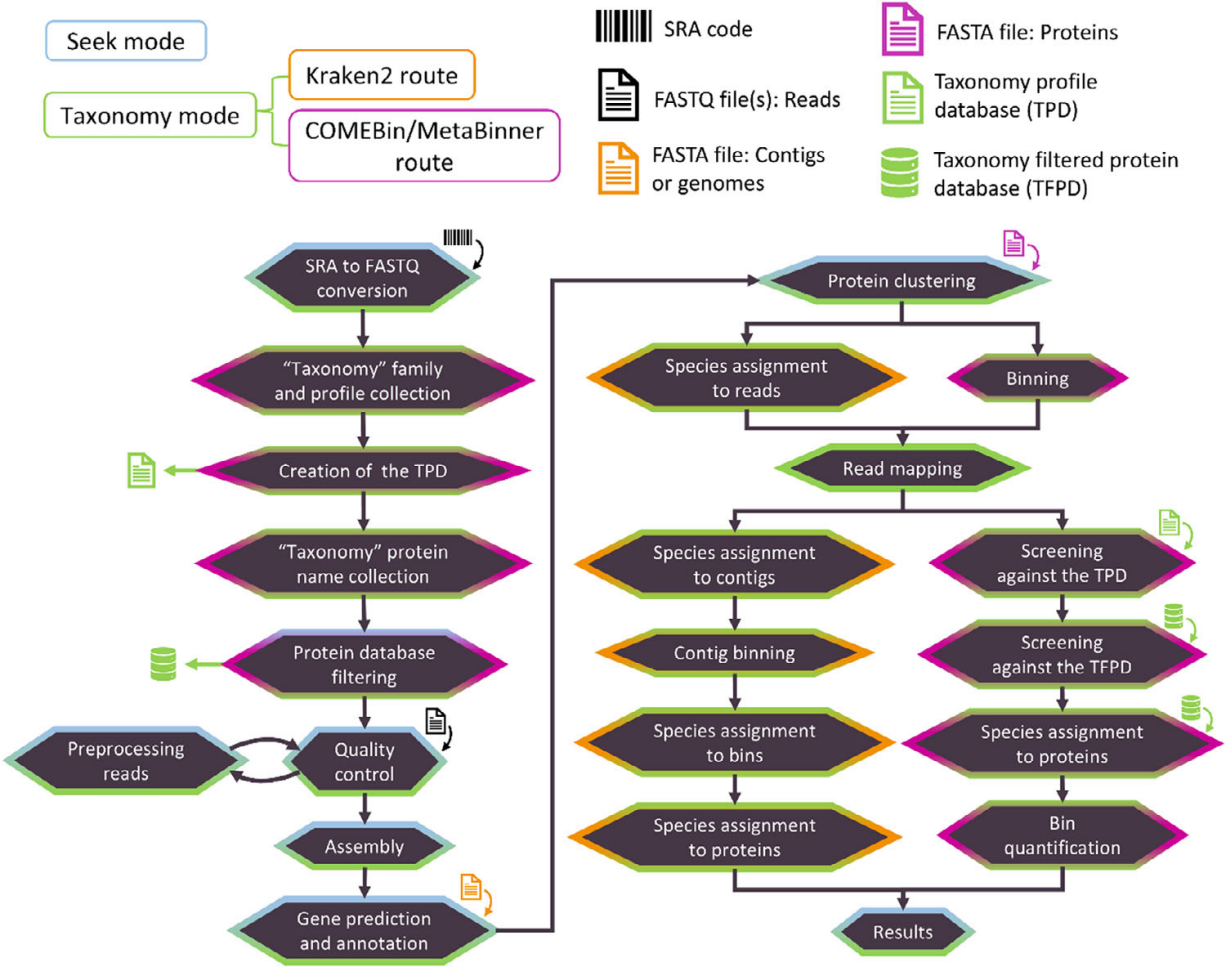
The stages of the “taxonomy” mode of *ProteoSeeker*. *ProteoSeeker* offers the functionalities of the “seek” mode (blue) and of the taxonomy mode (green). The taxonomic analysis is performed by the Kraken2 route (orange) or the COMEBin/MetaBinner route (purple). Each stage is colored based on the mode and route it belongs to. If an SRA code is provided the corresponding SRA and FASTQ files are generated. The “taxonomy” protein families are selected based on the input “taxonomy” family codes and their profiles are collected. The “taxonomy profile database” (TPD) is created. The “taxonomy” protein names of the selected families are collected, and the protein database is filtered based on these names creating the “taxonomy filtered protein database” (TFPD). The reads of the FASTQ files undergo several quality checks by FastQC. The reads are preprocessed by BBDuk and then reanalyzed by FastQC. The preprocessed reads are assembled into contigs by Megahit. Protein coding regions (pcdrs) are predicted in the contigs by FragGeneScanRs. CD‐HIT is used to reduce the redundancy of the pcdrs. For the Kraken2 route: Species are assigned to the reads based on Kraken2. Bracken then provides the abundances of these species. Bowtie2 maps the reads to the contigs. Species are assigned to contigs. The contigs are binned based on their species. Species are assigned to the bins. Species are assigned to the genes and proteins of the bins. For the COMEBin/MetaBinner route: The contigs are binned based on MetaBinner or COMEBin. Bowtie2 maps the reads to the contigs. The pcdrs are screened against the TPD with HMMER. Any pcdr with at least one hit against the TPD is screened against the TFPD with DIAMOND and any possible hit may provide one or more taxa whose TaxIds and lineages are found through TaxonKit. Taxa are then assigned to bins and to their genes and proteins. Each bin, along with any taxa assigned to it, is quantified. For both routes at the last stage annotation files are generated.

In addition, the taxonomy mode may be applied through two distinct routes. The “Kraken2 (taxonomy) route”, which includes the use of Kraken2,^[^ ^17^, ^18^ ^]^ Bracken and KrakenTools,^[^ ^19^, ^20^ ^]^ and the “COMEBin/MetaBinner (taxonomy) route”, which includes the application of COMEBin^[^ ^21^ ^]^ or MetaBinner.^[^ ^22^ ^]^ These routes cannot be combined in a single *ProteoSeeker* run, but due to the user's ability to initiate the run from different starting points in the pipeline, *ProteoSeeker* may perform taxonomic analysis starting from already assembled contigs. The analysis of Kraken2 and Bracken can be applied based on any Kraken2 and Bracken databases, respectively. The COMEBin/MetaBinner taxonomy route can be applied with any protein database that includes proteins with a header format that includes information about the organism according to the style used in the headers of the proteins in the non‐redundant (nr) database of NCBI^[^ ^15^, ^23^ ^]^ or the Uniref databases.^[^ ^24^, ^25^, ^26^, ^27^ ^]^ Any pre‐built or custom‐built Kraken2/Bracken or protein database apart from the default or proposed ones can be downloaded by the user and can be incorporated into the pipeline automatically, requiring as input the paths of these databases in the user's system. The stages of the pipeline in the taxonomy mode of *ProteoSeeker* are described in **Figure** **3**.

The specific individual tools incorporated in the *ProteoSeeker* pipeline were selected so as to integrate state‐of‐the‐art software for specific tasks. *ProteoSeeker* has been designed to perform efficiently in systems equipped with moderate RAM resources. Furthermore, each tool was appropriately chosen to be able to run in parallel processes or threads, leverage increased amounts of RAM when these are available, and ensure rapid analysis speeds compared to other tools with similar functionalities.

There are multiple options controlling the start and end points of *ProteoSeeker* to facilitate the application of the different seek or taxonomic analysis types and routes, allowing the user to omit prior stages of the pipeline or even subsequent ones. Hence, applying the seek or the taxonomy mode and a route with specific settings in a run can be based on previous analysis and does not require rerunning the whole analysis. Another time‐saving option of *ProteoSeeker* showcasing the versatility of the tool is the option to change the input Kraken2 or Bracken or protein database when reapplying *ProteoSeeker* against a dataset, with the run being initiated after the assembly or gene prediction or the binning stage.

*ProteoSeeker* is a Python command‐line tool and module, publicly available on GitHub^[^ ^28^ ^]^ and is also shipped as a Docker image in Docker Hub.^[^ ^29^ ^]^ For both forms, an analytical and straightforward installation manual is provided to the user at *ProteoSeeker's* GitHub repository and webpage. All *ProteoSeeker* relevant links, information, and instructions can be accessed through the tool's webpage.^[^ ^30^ ^]^

### 2.2 Associating Protein Families with Pfam Profiles and Protein Names

Specific stages of *ProteoSeeker* depend on the information acquired after processing the UniProtKB/Swiss‐Prot protein database.^[^ ^31^, ^32^, ^33^ ^]^ This information includes a file where each protein family identified in the latter database is represented by a specific protein family name. In addition, it offers the correspondence of each protein family name with a protein family code, the mean and median length (in number of amino acids) of its family members (proteins), and the protein IDs of the latter. For a family, each protein name is accompanied by its frequency, which is the number of times this name was found in the names associated with the proteins of the family. Also, another file contains each protein family represented by its family name. In addition, each family name includes a corresponding list of Pfam codes for Pfam profiles of the profile hidden Markov models (HMMs) associated with the family,^[^ ^34^, ^35^ ^]^ accompanied by their frequencies and a corresponding list of the UniProtKB/Swiss‐Prot protein IDs of the family members. This list of Pfam codes contains each set of Pfam codes that was computed to have the highest frequency amongst the sets for a given family. A set of Pfam codes based on a protein consists of each Pfam code corresponding to a domain found in the protein‐coupled with its frequency. Therefore, it is possible that a protein family may be associated with more than one set of Pfam codes, where all sets have the same frequency. It should be noted that region‐specific information about the similarity was taken into account when corresponding a protein to family names and codes (e.g., proteins with similarity based on the notes “In the C‐terminal section”, “In the central section” and “In the N‐terminal section” regarding the protein family resulted in distinct family names and codes) along with their related information. The scripts performing these analyses are available through *ProteoSeeker's* publicly available GitHub repository.^[^ ^28^ ^]^ Detailed information about the filtering process of a protein database can be found in the Supporting Information (Supporting Text: 1.2) of this article.

### 2.3 Experimental Validation of the Seek Mode

We validated the efficiency of the seek mode of *ProteoSeeker* in two distinct discovery expeditions. In both cases, the goal was to discover enzymes with specific functionality and, at the same time, to uncover candidate biocatalysts retaining enzymatic activity at predefined conditions to allow their incorporation into specialized industrial processes. In the first case, we targeted the discovery of thermostable amylolytic α‐amylase enzymes with optimal activity at 60–70 °C and neutral pH to be incorporated in industrial in‐line hydrolysis of starch‐rich preparations of infant foods. For this study, we selected as input various metagenomic datasets, both in‐house (datasets available via MG‐RAST associated with the sample names “HotZyme Ch2‐EY65S” and “HotZyme sun spring”) and publicly available ones (SRA code: SRR17771278), with near‐neutral pH values and sampling temperatures ranging from 60 to 70 and ≈50 °C, respectively. Part of these datasets originate from the previously EU‐funded project HotZyme (https://cordis.europa.eu/project/id/265933/reporting), in the framework of which numerous biotechnologically valuable novel enzymes have already been discovered.^[^ ^5^, ^36^, ^37^, ^38^ ^]^

*ProteoSeeker* analysis led to the identification of proteins within a timeframe ranging from a few minutes to a few hours, depending on the analyzed metagenomic dataset and desired protein function. This showcases that the tool offers fast analysis, a feature which can be attributed to its automation and utilization of the carefully selected set of analysis tools in its pipeline and their time‐efficient analysis (e.g., BBDuk, MEGAHIT, FragGeneScanRs, CD‐HIT, DIAMOND, HMMER, Kraken2, Bracken). Consequently, when enough RAM and CPUs are available to run an analysis, *ProteoSeeker* offers a rapid approach to discovering and annotating putative proteins compared to other available tool combinations. Time‐efficiency is enhanced further due to the availability of multiple options and processes, which have been automated to facilitate the running of *ProteoSeeker* by non‐expert users. Detailed execution times for the runs that led to experimentally verified proteins can be found in the Supporting Information (Supporting Text: 1.3).

*ProteoSeeker* identified ≈800 000 protein‐coding regions from these samples. For the in‐house samples, 299 protein‐coding regions included at least one domain associated with α‐amylases (e.g., profiles with the short names of “Alpha‐amylase”, “Alpha‐amylase_C”, “Alpha‐amylase_N”, “Alpha‐amyl_C2”) and were fully annotated by *ProteoSeeker*. 108 out of these 299 proteins contained at least one “Alpha‐amylase” domain, did not include signal peptides and their best match against the UniProt/Swiss‐Prot database did not have an approximate percentage identity above 80%. The annotation output file for these 108 sequences is provided in Table S2 (Supporting Information). Please note that the headers of the annotation file were renamed and columns removed to better align with the current output version of *ProteoSeeker*. None of these modifications relates to the information based on which the filtering criteria were applied. To further narrow down the selection, the difference in sequence length of each putative protein with the mean length of the members of the “glycosyl hydrolase 13 family (GH13)” was taken into consideration. GH13 was selected as a reference as it includes enzymes involved in the breakdown of substrates containing α‐glucoside linkages. Amylases are one of the major subgroups within this family.^[^ ^39^ ^]^ The mean length of the family was computed as the mean of the lengths of the proteins from the UniProtKB/Swiss‐Prot database belonging to the GH13 family. We focused on proteins with small differences in length with the mean length of the GH13 family. In addition, other desirable characteristics were the presence of start and stop codons in the sequence of the putative protein and a small identity percentage with the best match of the protein against the UniProtKB/Swiss‐Prot database. Instead of sequentially filtering proteins by applying each criterion separately (e.g., first filtering by length, then by the presence of start and stop codons, etc.), we evaluated all criteria simultaneously for each candidate protein and only retained those that met all the conditions. Consequently, two sequences (AL_6, AL_15) from the filtered 108 putative proteins were randomly selected as candidates for experimental evaluation. Based on the analysis of the publicly available metagenomic dataset, *ProteoSeeker* identified 723 protein‐coding regions, which included at least one domain associated with α‐amylases. These regions were, thus, fully annotated. Similarly to the in‐house samples, the same criteria regarding the length of the proteins, the presence of at least one “Alpha‐amylase” domain, the presence of start and stop codons, and a small identity percentage with the protein's best match against the UniProtKB/Swiss‐Prot database, regardless of the absence of a signal peptide in this case, were applied. Based on these criteria, from the filtered 723 annotated proteins, one sequence (AL_17) was randomly selected for experimental validation. Overall, the results provided by *ProteoSeeker* allow for variable criteria application based on the end‐goal of the user. This approach highlights the utility of the tool's output and accompanying information, showcasing the versatility and ease of maximizing the chances of selecting protein sequences likely to exhibit the desired activity.

Following candidate selection, the DNA sequences encoding the putative proteins were codon‐optimized for recombinant production, overexpressed in *Escherichia coli*, and purified. Their biochemical characterization revealed that all selected proteins (AL_6, AL_15, AL_17) exhibit amylolytic activity within the targeted temperature and pH range (Figure S1, Supporting Information). The nucleotide sequence of the gene encoding the AL_17 enzyme reported herein is available in the Third‐Party Annotation (TPA) Section of the DDBJ/ENA/GenBank^[^ ^40^, ^41^, ^42^ ^]^ databases under the accession number TPA: BK068330.

Subsequently, *Proteoseeker* was applied for the targeted discovery of carbonic anhydrases (CAs) for industrial CO_2_ capture applications. The discovery of such biocatalysts is rather challenging as the enzyme is intended for industrial CO_2_‐capture applications and is required to perform under hot potassium carbonate (HPC) conditions, which include temperatures above 80 °C and pH of 11.5. These stringent biochemical requirements necessitate exquisite thermostability and activity under high alkalinity, which are exceptionally rare characteristics for natural proteins. By utilizing these criteria to guide metagenomic dataset selection, the utilization of *Proteoseeker* led to the discovery of CA‐KR1,^[^ ^43^ ^]^ a novel CA exhibiting unprecedented stability and tailored biochemical characteristics that meet the demands of industrial CO_2_ capture pipelines. The discovery of CA‐KR1, which is, to the best of our knowledge, the most stable CA known to function under HPC conditions, highlights the great potential of *ProteoSeeker* for discovering new useful proteins. To identify CA‐KR1, *ProteoSeeker* analyzed 28 metagenomic datasets, which were selected based on their annotated sample collection temperatures and pH values. The threshold of minimum temperature for the selection of the datasets was 80 °C to accommodate industrial application conditions.

A total of ≈100 000 protein‐coding regions were predicted during the runs of *ProteoSeeker* for the 3 datasets (SRA codes: DRR163688, SRR3961740, SRR14762249) corresponding to the highest collection temperatures based on their environmental samples. From these protein‐coding regions, *ProteoSeeker* predicted 31 proteins with domains corresponding to profiles associated with CAs through its type 1 analysis of the seek mode. Through the type 2 analysis of the seek mode, *ProteoSeeker* detected ≈76 proteins having at least one hit with an E‐value equal to or below 1e‐70 through DIAMOND against the “seek filtered protein database” (SFPD). Manual analysis of the output information provided by *ProteoSeeker*, including evaluation of the output data as mentioned above for the amylases (protein length, start and stop codon presence, etc.) led to the selection of nine proteins to be experimentally tested. The coding DNA sequences of the latter proteins were cloned and expressed in *E. coli*. Out of them, three proteins, CA‐KR1,^[^ ^43^ ^]^ CA_89, and CA_201 (unpublished) were produced at a level sufficient for biochemical characterization and were found to exhibit CA activity, thus showcasing again the value of *ProteoSeeker* for new biotechnological discoveries. The nucleotide sequences of the genes encoding the CA‐KR1 and CA_201 enzymes reported herein are available in the TPA Section of the DDBJ/ENA/GenBank databases under the accession numbers TPA: BK065798 and TPA: BK068331, respectively. Further information regarding the biochemical characterization of CA‐KR1 can be found in the work of Rigkos et al.^[^ ^43^ ^]^

### 2.4 Evaluation of the Taxonomy Mode

To analyze and evaluate the different taxonomy routes of *ProteoSeeker*, nineteen SRA files were analyzed. These files correspond to the “gold standard” samples of artificial (simulated) nature and samples originating from cultures of known species compositions. The same files and the methodology followed to create them are described in the work of Poussin et al. (Supporting Text: 1.4, Supporting Information).^[^ ^44^ ^]^ *ProteoSeeker* version 1.0.0 was used for the evaluation. Each gold standard dataset contains a specific number of species that approximates one of the following numbers: 10, 40, 120, 500, and 1000. Hence, each sample has been categorized based on the closest approximation of these numbers, forming four groups of three samples each, and one group of seven samples. The latter group corresponds to the group of ten species, and it includes four samples originating from ZyMoBIOMICS cultures.^[^ ^44^ ^]^ Each group contains three samples of artificial origin, one with no bias, one being AT‐rich biased and one being GC‐rich biased in terms of DNA base content (Table S3, Supporting Information).

Initially, each of the samples above was downloaded and processed from *ProteoSeeker* using its SRA code as input. Then, *ProteoSeeker* analyzed each sample through five different runs, each time utilizing a different taxonomy route or protein database or Kraken2 / Bracken database. As a result, three runs used the Kraken2 taxonomy route based on the Kraken2 / Bracken Refseq indexes of the Standard‐8, Standard‐16, and Standard collections,^[^ ^45^ ^]^ which are databases, size 8 GB, 16 GB, and 77 GB, respectively. For each of these runs, Bracken was applied to estimate the abundances of the species predicted by Kraken2 by filtering out each species with reads less than 10 and by targeting the species level. A “taxonomy filtered protein database” (TFPD) was created from the nr database in a *ProteoSeeker* run, independently of the evaluation analysis, and was based on protein families associated with RNA polymerases (“RNApol TFPD”). One run used the COMEBin/MetaBinner taxonomy route, utilizing COMEBin, and was based on the RNApol TFPD. Another run used the COMEBin/MetaBinner taxonomy route, utilizing MetaBinner, and was based also on the RNApol TFPD. Each run generated a set of predicted species accompanied by their abundances or/and relative abundances. These results of each run correspond to the sample analyzed by *ProteoSeeker* in the run. The Kraken2 taxonomy route allows for the use of multiple threshold values for the filtering step of the species after Bracken has been applied. For the purposes of the evaluation, the threshold values applied were 0.01%, 0.1%, 1.0%, 5.0%, 100, 500, and 1000, and values for the thresholds automatically computed by *ProteoSeeker* based on the Shannon index^[^ ^44^, ^46^, ^47^ ^]^ value for the case of “non‐gut” and “gut” samples, according to the methodology described by Poussin et al.^[^ ^44^ ^]^ The Shannon index was computed based on KrakenTools.^[^ ^19^ ^]^ In addition, there was a case where no filtering threshold was applied. In total, nine non‐zero filtering thresholds were used for the evaluation. The annotation files contain binning and taxonomy information based on the species remaining after filtering the species of Bracken based on a specific filtering threshold (which can be user‐defined). Bracken was applied with a read length equal to 100, which is the closest value to the average length of the reads in each sample, and with a threshold equal to 10 reads as proposed by the protocol described by Lu et al. to reduce low‐abundance noise.^[^ ^19^ ^]^ The COMEBin/MetaBinner taxonomy route allows for more than one taxa to be associated with a bin, in which case they also share the same abundance and relative abundance. The automatically computed Shannon index and filtering threshold values for non‐gut and gut samples based on each Kraken2 database and sample are provided in Table S4 (Supporting Information). Each gold standard dataset was run by *ProteoSeeker*, and its results were evaluated based on the known species and relative abundances for the gold standard dataset. The metrics used to perform the evaluation were the “True Positive (TP)” hits, “False Positive (FP)” hits, “False Negative (FN)” hits, “Sensitivity”, “Precision”, “Accuracy”, “F1 Score”, “Jaccard Index” and “L1 Norm”.^[^ ^44^, ^48^, ^49^, ^50^ ^]^ More information for the metrics can be found in the Supporting Information of this article (Supporting Text: 1.5).

The evaluation of the taxonomy mode took advantage of the stage initiation options of *ProteoSeeker*. Before any of the runs were executed, different procedures of *ProteoSeeker* were performed individually: the creation of the profile databases, the filtering of the protein database, and the collection and processing of each SRA dataset. These operations were performed separately to showcase that the procedures related to processing an SRA code and creating the profile and filtered protein databases can be performed once. After that, they can be used directly by any subsequent *ProteoSeeker* run for either the seek or the taxonomy mode. Hence, the SRA dataset is downloaded and processed once, and then each subsequent *ProteoSeeker* run recognizes the presence of the SRA sample locally and utilizes it directly (similarly for the profile and filtered protein databases). The creation of the profile database and filtered protein database based on nr, by *ProteoSeeker* took ≈38 min to complete, utilizing 16 CPUs. Processing an SRA dataset involves converting the SRA file to one or more FASTQ files. *ProteoSeeker* examines whether the output FASTQ files are paired‐end, single‐end or both and proceeds accordingly.

The first run of each sample analysis used the Kraken2 taxonomy route and the Standard‐8 collection as a database. The following two runs of the same route initiated *ProteoSeeker* after the stage of gene prediction and used the Standard‐16 and Standard collections as databases, respectively. Then, the COMEBin/MetaBinner taxonomy route was used, with COMEBin, where *ProteoSeeker* started the analysis after the stage of gene prediction. The latter procedure was followed also for the COMEBin/MetaBinner taxonomy route, with MetaBinner. The taxonomy routes, route‐specific tools, and databases used in the evaluation can be found in Table S5 (Supporting Information). All runs of *ProteoSeeker* were performed in an Ubuntu 24.04 LTS system with 124 GB of RAM and 32 CPUs available. The general thread option of *ProteoSeeker* (“‐t/–threads” option) was set equal to 24, so every tool or custom process in its pipeline utilized up to 24 processes or threads‐if that tool offered such an option. Specifically, the option for the threads regarding the creation of the profile and filtered protein databases (“‐ft/–filtering‐threads” option) was set equal to 16. In the Kraken2 taxonomy route, Kraken2 was not applied with memory mapping, and in the COMEBin/MetaBinner taxonomy route, COMEBin utilized an NVIDIA GeForce RTX 4090 GPU. In addition, only contigs with a length higher than 1000 nucleotides were used in the binning processes of COMEBin and MetaBinner. The parameter files and test scripts used to perform the evaluation can be found in the GitHub repository of *ProteoSeeker* which is publicly available.^[^ ^28^ ^]^

**Figure 4:**
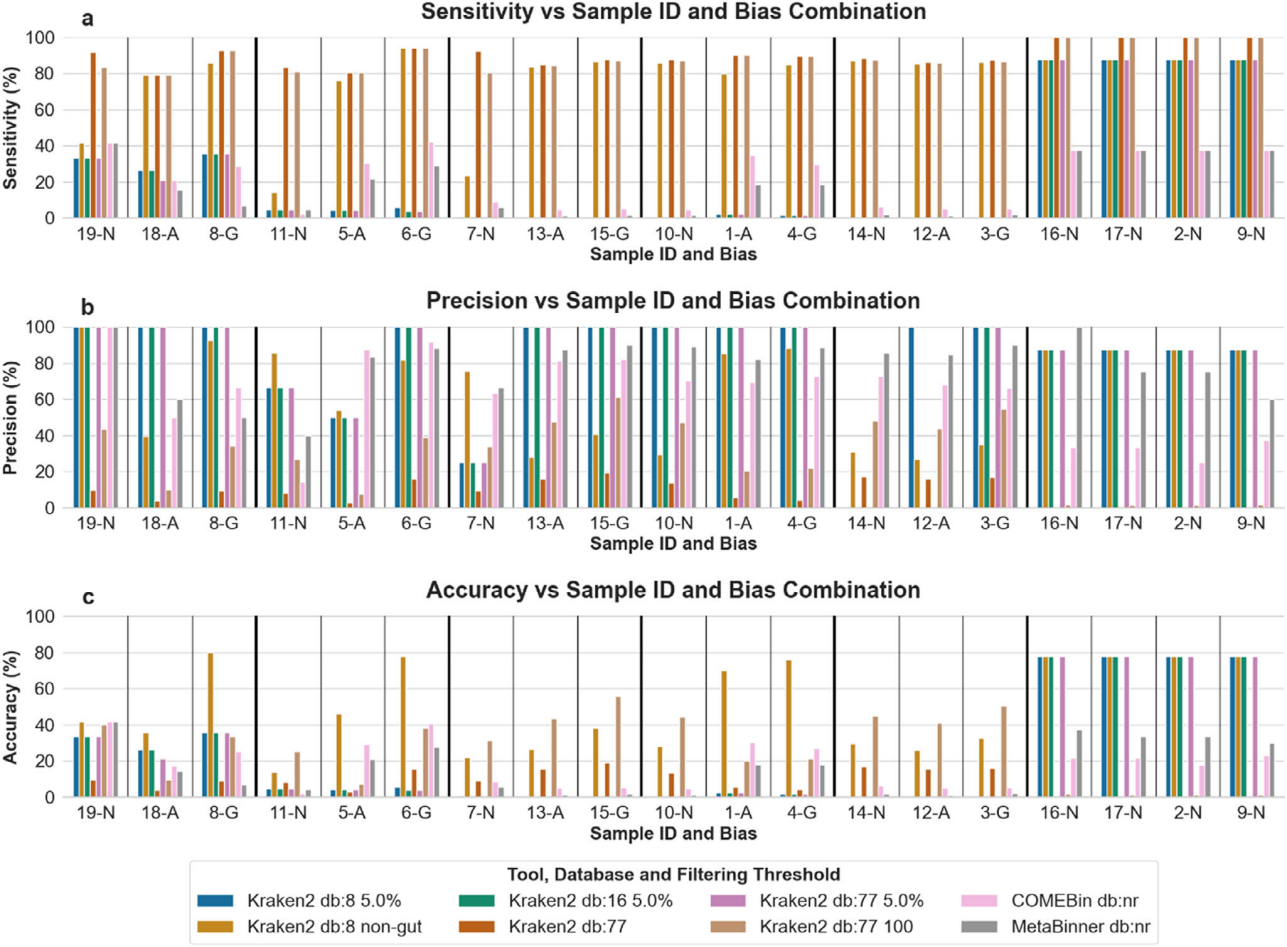
Sensitivity a), precision b), and accuracy c) of the taxonomy mode evaluation results, with the samples sorted based on their species‐abundances and biases. The results were acquired by the *ProteoSeeker* runs for the selected combinations of Kraken2 databases and filtering thresholds plus the COMEBin/MetaBinner taxonomy methods for each sample of the 19 gold standard datasets. The selected combinations regarding the Kraken2 taxonomy route include the database of the Standard‐8 collection with the filtering threshold of 5.0% (“Kraken2 db:8 5.0%”) and the filtering threshold computed based on non‐gut samples (“Kraken2 db:8 non‐gut”), the database of the Standard‐16 collection with the filtering threshold of 5.0% (“Kraken2 db:16 5.0%”) and the database of the Standard collection without a filtering threshold (“Kraken2 db:77″) and with the filtering thresholds of 5.0% (“Kraken2 db:77 5.0%”) and of 100 (“Kraken2 db:77 100″). The COMEBin/MetaBinner taxonomy route was applied through COMEBin with the nr protein database as the filtering target (“COMEBin db:nr”) and through MetaBinner with the nr protein database as the filtering target (“MetaBinner db:nr”). The samples are sorted into groups of species‐abundances. Samples 19, 18, and 8 for 10 species from simulated reads, samples 11, 5, 6 for 40 species, samples 7, 13, 15 for 120 species, samples 10, 1, 4 for 500 species, samples 14, 12, 3 for 1000 species and samples 16, 17, 2, 9 for 10 species from cultures. The letters “N”, “A” and “G” on the labels stand for “No bias”, “AT‐rich bias” and “GC‐rich bias”, respectively.

The results were initially processed according to all filtering thresholds applied in the Kraken2 taxonomy route, plus the case of not applying a filtering threshold, and all Kraken2 databases used in the runs (Table S5, Supporting Information). The best scoring combinations of Kraken2 databases and filtering thresholds or no threshold, based on each metric, were used in evaluating the results collected from the COMEBin/MetaBinner taxonomy route with COMEBin and MetaBinner based on the nr protein database (**Figure** **4**; Figures S2 and S3, Supporting Information). These combinations include the database of the Standard‐8 collection with a filtering threshold of 5.0% and the filtering threshold computed for non‐gut samples, the database of the Standard‐16 collection with the filtering threshold of 5.0% and the database of the Standard collection without a filtering threshold and with the filtering thresholds of 100 and of 5.0%. The best scoring combinations for a metric are the combinations with the same highest frequency for that metric, based on their scores across the nineteen gold standard samples. The frequency of a combination is equal to the number of samples (out of the 19) in which it achieves the best score. The best score is either the highest (for true positive hits, sensitivity, precision, accuracy, F1 score, Jaccard index) or the lowest (for false positive hits, false negative hits, L1 norm) score depending on the metric. The frequency of the best scoring combinations for each metric based on all 19 samples is found in Table S6 (Supporting Information).

**Figure 5:**
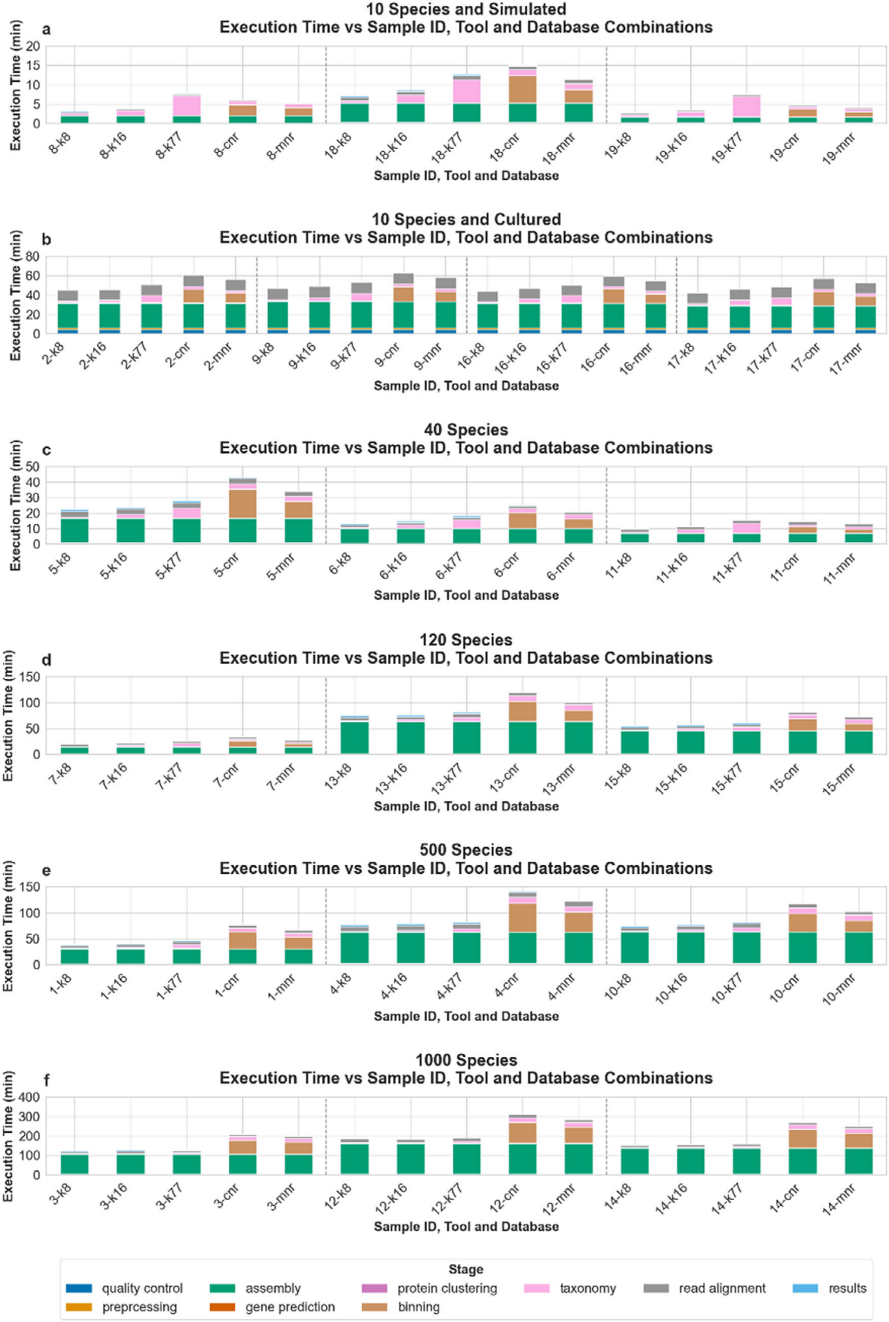
Stage‐specific execution times of *ProteoSeeker* runs for each sample, tool, and database. Each bar is labeled as “sample ID‐tool‐database”. For each sample, the times related to the runs with the Kraken2 taxonomy route and databases of the Standard‐8 collection (“k8”), Standard‐16 collection (“k16”), and Standard collection (“k77”), plus the COMEBin/MetaBinner taxonomy route with COMEBin (“cnr”) and MetaBinner (“mnr”) with the nr database are shown. The stage of creating the profile and filtered protein databases and the stage of collecting and processing each SRA sample are excluded from the time analysis shown in the plots. The stages up to and including the stage of gene prediction share the same execution times for all methods of each sample. The plots have been divided into 5 categories, one for the group of 10 species that originate from simulated reads a), one for the group of 10 species originating from cultures b), one for the group of 40 species c), one for the group of 120 species d), one for the group of 500 species e), and one for the group of 1000 species f). The execution times of *ProteoSeeker* were based on the evaluation of its taxonomy mode. The evaluation was based on running *ProteoSeeker* in an Ubuntu 24.04 LTS system with 124 GB of RAM, 32 CPUs, and an NVIDIA GeForce RTX 4090 GPU available. For these runs *ProteoSeeker's* general thread option (“‐t/–threads” option) was set equal to 24, so every tool or custom process in its pipeline utilized up to 24 processes or threads – if that tool offered such an option. Kraken2 was not applied with memory mapping and in the COMEBin/MetaBinner taxonomy route, COMEBin utilized the GPU of the system. In addition, only contigs with a length higher than 1000 nucleotides were used in the binning processes of COMEBin and MetaBinner. The parameter files and test scripts used to perform the evaluation can be found in the GitHub repository of *ProteoSeeker*.

The time needed to apply Bracken and filter the report of Bracken is negligible compared to the time needed for the analysis to take place (<1 min) as shown also by the work of Lu et al.^[^ ^19^ ^]^ The computed execution time of *ProteoSeeker* for the taxonomy evaluation did not include the application of Bracken and the subsequent filtering process, although it included applying the filtering thresholds directly to the results of Kraken2. The time difference in filtering the results of Bracken instead of Kraken2 is negligible. The execution time was also based on the post‐classification taxonomy‐related processes (e.g., binning, annotation file generation) of the species filtered from the output of Kraken2 based on a specific filtering threshold. Therefore, while the reported execution times do not include the Bracken analysis and are based on selecting the species from the Kraken2 output, they form a solid basis to compare the time efficiency of the different combinations of taxonomy routes and databases and of their individual stages in the pipeline. In general, Bracken is applied rapidly, the filtering thresholds are applied to its output in an identical way as to the output of Kraken2 and the filtered species consist of an unknown set of species a priori, which is determined based on the abundance of species and one specific filtering threshold. Consequently, the computed execution times are a representative example of how the execution time of *ProteoSeeker* behaves in each stage and how it differs between the two taxonomy routes and between the use of different databases in the Kraken2 taxonomy route based on the taxonomy evaluation (**Figure** **5**).

The results of the COMEBin/MetaBinner taxonomy route for each of the two binning tools may include multiple taxa associated with one bin ID and one relative abundance. Based on the statistical analysis of the results, which is not included in the pipeline of *ProteoSeeker*, only species associated with bins and their relative abundances were considered for each sample. In addition, the species associated with bin IDs, which numbered more than 1, were discarded. This allowed us to make a more stringent selection of the species that were identified and associated with bin IDs and thus each sample. A species solely associated with more than one bin ID receives a relative abundance equal to the sum of the relative abundances of its associated bin IDs. This type of filtering for the species predicted by the COMEBin/MetaBinner taxonomy route can be performed through an additional script available in the GitHub repository of *ProteoSeeker*.^[^ ^28^ ^]^ The same script, apart from generating a new filtered species report can also generate a new TXT annotation file updated based on the filtered species and merged bin IDs. The bin IDs of the same species each of which is assigned only one species are “merged” as their relative abundances for their common species are summed up.

To achieve comparable total execution times for the different *ProteoSeeker* runs in the evaluation of the taxonomy mode, specific adjustments were needed due to the varying stage initiation processes between the runs. When computing the stage‐specific and total execution time of the *ProteoSeeker* runs, the execution time for the initial stages of the pipeline was acquired by the first run of the tool, which was the run for the Kraken2 taxonomy route with the Standard‐8 collection as a database. These initial stages refer to the stages of read quality check (quality control), read preprocessing, read assembly, and gene prediction.  The execution times for the latter stages were added to the runs that did not include them. This led to the runs having the same execution times for these initial stages of the pipeline. The total and stage‐specific execution times of *ProteoSeeker* gave us an insight into which stages act as the bottleneck in the analysis and formed the basis for understanding how *ProteoSeeker* may be improved in the future in terms of speed (Figure 5).

In addition, the execution time of *ProteoSeeker* was analyzed based on the size of the dataset under analysis and the number of species in that dataset for each combination of taxonomy method and database (Figures S4 and S5, Supporting Information). Specifically, for the case of the species abundance, the mean time was computed for each sample category of species abundances (10, 40, 120, 500, and 1000) as well as for the two subgroups of the samples with 10 species which originate from simulated reads and cultures, respectively.

### 2.5 Taxonomy Mode Evaluation for Proteins of Expected Protein Families

The taxonomy mode of both routes was also applied to the FASTQ samples, in which the novel AL and CA enzymes AL_17, CA‐KR1, and CA_201 were identified. Based on our search for novel ALs and CAs with specific biochemical characteristics, five enzymes were experimentally profiled. Two of these enzymes (AL_6, AL_15) were identified by running *ProteoSeeker* on a file with contigs, where the taxonomy routes cannot be applied. Thus, each of the three enzymes CA‐KR1, CA_201, and AL_17 was used to compare taxonomy results among the Kraken2 and the COMEBin/MetaBinner taxonomy routes of *ProteoSeeker*, and the taxonomy classification of the best hit from the BLASTP results acquired using the NCBI's online blastp suite against the nr database with the default parameters.^[^ ^51^, ^52^ ^]^ The latter results can be found in Table S7 (Supporting Information). In both cases of mining for ALs or CAs, the protein families selected for the taxonomy route of COMEBin/MetaBinner were associated specifically with the targeted protein family. *ProteoSeeker* was run on the same system as the one used to run the taxonomy evaluations based on the gold standard samples. While using the seek mode of *ProteoSeeker* to search for proteins in the family of β‐CAs, the taxonomy route of COMEBin/MetaBinner was applied based on different protein families (e.g., α‐, β‐ and γ‐CA families) and protein names associated in general with CAs. Similarly, while searching for proteins in the protein family of α‐amylases, the taxonomy route of COMEBin/MetaBinner was applied based on different protein families and protein names associated in general with ALs. The COMEBin/MetaBinner taxonomy route was applied using COMEBin and MetaBinner with the nr database as the protein database to be filtered and the Kraken2 taxonomy route was applied based on the databases of the Kraken2/Bracken Refseq indexes of the Standard‐8, Standard‐16 and Standard collections. For either one of the routes, no filtering threshold was applied to the predicted taxa.

## 3 Discussion

### 3.1 *Proteoseeker* Implemented as a Command‐Line Tool Offers Accessible Protein Discovery and Annotation from W(M)GS, Genomic and Proteomic Data

*ProteoSeeker* identifies proteins by automatically processing W(M)GS data (e.g., environmental metagenomes), as well as genomic or proteomic data. It can then focus on screening the identified proteins based on selected protein families and offering taxonomy prediction of the organism(s) associated with each protein.

*ProteoSeeker* provides several advantages over other metagenomic analysis pipelines. *ProteoSeeker* has been implemented as a command‐line tool and module developed in the Python programming language, is publicly available in GitHub^[^ ^28^ ^]^ and is also shipped as a Docker image, available publicly in Docker Hub.^[^ ^29^ ^]^ The installation of *ProteoSeeker* and its utilization via its Docker image are straightforward and detailed instructions for both can be found in *ProteoSeeker's* GitHub repository. It is a comprehensive tool, designed to achieve specific objectives, suitable for use by both non‐experts and more advanced users. It can handle different kinds of inputs, including W(M)GS data, contigs, genomes, and proteomic datasets, as well as SRA codes from the SRA database of NCBI.

The pipeline of *ProteoSeeker* is designed with a focus on time efficiency and compatibility with systems possessing moderate RAM and CPU resources. However, it can effectively utilize all such available resources when needed. Its pipeline incorporates a suite of state‐of‐the‐art tools and provides multiple configurable options for customization. The latter characteristics were prioritized over disk space. Scalability was another important criterion, focusing on the tools' ability to handle large datasets, with equal proficiency as smaller ones, when utilizing more CPUs or RAM. When sufficient disk space is available for installing *ProteoSeeker* and its necessary databases, RAM becomes the bottleneck for Kraken2, while the number of CPUs determines performance for most other tools.

The functionalities offered by *ProteoSeeker* primarily involve identifying proteins from the input data, screening the proteins according to user‐defined protein family codes and/or protein names, and performing taxonomic analysis of the proteins. All functionalities are highly customizable and can be performed either independently or collectively in a single run. The capabilities of *ProteoSeeker* are ideal for analyzing metagenomic datasets of low or high complexity and provide annotation for the proteins identified as well as a series of output files that include multiple kinds of information (e.g., quality control results for the reads, preprocessed reads, protein‐coding regions, all putative proteins, binning information, taxonomy classifications of the reads and bins).

The tool's functionalities are divided into two main modes of function, each mode including its own types and routes of analysis. Users can control the functions of the tools in the pipeline and the analysis process through the options provided by *ProteoSeeker*. *ProteoSeeker* can be run with minimal input requirements, needing only an SRA code or FASTQ/FASTA file(s) as input, and when necessary, selecting one or more protein families of interest. The output of *ProteoSeeker* is also user‐input dependent, allowing the results to be documented according to whether the user prefers to include information from the seek mode, the taxonomy mode, or both.

One aspect to consider regarding the seek mode of *ProteoSeeker* is that it should be able to adequately recognize each protein family represented by a single Pfam profile, unique to the family. Selecting a protein family with at least one domain not unique to the family will lead *ProteoSeeker* to search for all families associated with that domain. While a protein belonging to such a protein family should be identified by *ProteoSeeker*, it could be mixed with others, each comprising a separate set of domains that includes at least one associated with the selected protein families. Despite this, the additional information provided by *ProteoSeeker* facilitates the process of discarding certain proteins. For example, such a helpful step in the latter process is the evaluation of the protein length of each of the identified proteins compared to the protein family of its best hit against the UniProtKB/Swiss‐Prot database and the provided mean length of the latter family. In addition, the presence or absence of signal peptides and transmembrane domains is another piece of information that might help the user decide whether a predicted protein belongs to the targeted protein family. Furthermore, a protein identified by *ProteoSeeker* after successfully passing the profile screening stage will contain at least one domain corresponding to the selected protein families, though not necessarily all. Protein families not represented by any profile in Pfam remain a blind spot in *ProteoSeeker's* screening during type 1 analysis of the seek mode.

### 3.2 *Proteoseeker's* Seek Mode Evaluation is Showcased by Application‐Driven Enzyme Discoveries Including a Biocatalyst for Industrial Carbon Capture

The usefulness of *ProteoSeeker* in effectively identifying proteins with targeted functionalities, based on known protein families, has been demonstrated by discovering novel amylases and carbonic anhydrase enzymes with targeted biochemical characteristics. Notably, the discovery of CA‐KR1^[^ ^43^ ^]^ highlights a high‐profile enzyme with significant potential for carbon capture applications, gathering considerable attention from the broader community.^[^ ^53^, ^54^, ^55^, ^56^, ^57^ ^]^ Both cases of enzyme discovery demonstrate the power of *ProteoSeeker* when its capabilities are synergized with the analysis of metagenomic samples based on the search for proteins with specific characteristics according to their Pfam domains and by extension their protein families. This search is supported by allowing the user to provide one or more protein family codes, protein names, and protein databases to *ProteoSeeker* as input.

Furthermore, the evaluation of the seek mode of *ProteoSeeker* underlines another important observation which is that *ProteoSeeker* may simply be used for the discovery of protein sequences based on the input dataset. A user does not have to provide protein family codes, protein names, or protein databases to *ProteoSeeker* if the end‐goal of the analysis is to acquire a set of protein‐coding regions originating from the input data. Such an analysis is generally common and the pipeline of *ProteoSeeker* is suitable to perform it. In addition, a set of proteins can be used as input to *ProteoSeeker* directly in order to get as output their annotation.

Hence, a user may combine the automation provided by *ProteoSeeker* and the information available for a (meta)genomic dataset to acquire either just a set of protein sequences originating from the dataset or proteins of certain desirable characteristics (e.g., functionality, evolutionary origin).

### 3.3 *Proteoseeker's* Taxonomy Mode Evaluation Offers a Direct Comparison of Both Taxonomy Routes and an Insight into the Effect of Different Combinations of Kraken2/Bracken Databases and Filtering Thresholds

The evaluation of the taxonomy mode of *ProteoSeeker* was based on artificially created (simulated) reads and on reads originating from known mixtures of microorganisms (cultures), with the addition or not of specific biases. The protein database selected to be used in the COMEBin/MetaBinner taxonomy route was the nr database. Other elements were considered in the evaluation, such as the database used in the Kraken2 taxonomy route, as well as the different filtering thresholds applied to the species identified by Kraken2 and processed by Bracken. The results of the evaluation offered a series of interesting observations. The evaluation results were studied according to the scores of the metrics for the different routes, tools, databases, and filtering thresholds and in regard to the biases and origin of the samples (Figure 4; Figures S2 and S3, Supporting Information). An initial observation is that the scores of the same combinations of taxonomy routes, tools, databases, and filtering thresholds seem to follow the same pattern between the different samples of each species‐abundance category. This pattern seems to be most disrupted for the two groups of low‐species numbers, meaning 10 and 40, and to be most evident for the groups containing high numbers of species and especially for the “control” samples originating from cultures. This is an expected outcome since samples containing a few species, based on a few differences regarding their true positive, false positive, and false negative hits, will show larger discrepancies between their scores regarding the rest of the metrics, compared to samples containing higher numbers of species. The same applies to the L1 norm metric, which also accounts for the relative abundances of the species. In addition, the validation group of species and their relative abundances, based on each of the control samples, is the same and, therefore, the results of the taxonomy routes were expected to be the most similar for these samples. In addition, most combinations of Kraken2 databases and filtering thresholds, for most samples, showed increased sensitivity, precision, and accuracy for the GC‐rich samples compared to the same samples with no bias and with AT‐rich bias. The results from the COMEBin/MetaBinner taxonomy route did not reveal any evident pattern regarding the biases of the samples.

Furthermore, based on the best scoring combinations of Kraken2 databases and filtering thresholds (Kraken2 combinations) is that the Standard‐8 and Standard‐16 collections generally provide adequate results and, in some cases, the best outcomes when combined with the appropriate filtering thresholds, even surpassing the Standard collection. Several combinations of these databases with different filtering thresholds were present in the top 2–3 combinations for different metrics, as shown in Table S6 (Supporting Information). This is a crucial observation because the Standard‐8 and Standard‐16 collections do not require large amounts of disk space as the Standard collection or other databases demand.

The selected best‐scoring Kraken2 combinations, based on each metric, involve the database of the Standard‐8 collection with the filtering threshold of 5.0% and the filtering threshold computed for non‐gut samples, the database of the Standard‐16 collection with the filtering threshold of 5.0% and the database of the Standard collection without a filtering threshold and with the filtering thresholds of 100 and 5.0%. Of all combinations, the Standard‐8 collection combined with the filtering threshold computed for non‐gut samples is for most samples in the top 2 or 3 scoring methods offering a consistent performance across all metrics, except for precision. The same combination has the best performance for accuracy for several samples. The Standard‐8 collection with the filtering threshold computed for non‐gut samples and the Standard collection without a filtering threshold and with a filtering threshold of 100 has the best performance for sensitivity. The Standard‐8, Standard‐16, and Standard collections, each with a filtering threshold of 5.0%, have the best performances for most samples for precision. Furthermore, the combinations of the Standard‐8 collection with the filtering threshold computed for non‐gut samples and the Standard collection with the filtering threshold of 100 are in the top 2–3 scoring combinations for several samples based on the F1 score, Jaccard index, and L1 norm. The latter metric is the one that also accounts for the relative abundance of the species.

While computing the different metrics, the species taken into account by the COMEBin/MetaBinner taxonomy route come from the bins associated with one species each. The relative abundance of each of these species is the sum of the relative abundances of the same species based on all bins that were associated with that species alone. The COMEBin/MetaBinner taxonomy route by analyzing solely its own performance, scored in general low for sensitivity and accuracy and much higher for precision. Based on the F1 score and Jaccard index, COMEBin and MetaBinner scored moderately and based on the L1 norm they scored poorly relative to the Kraken2 taxonomy route.

In general, it should be noted that the combinations of the Kraken2 databases of the Standard‐8, Standard‐16, and Standard collections with the 5.0% filtering threshold seem to have very similar scores in all metrics and samples. A closer examination of the results shows that the number of species predicted by Kraken2 in each sample increases with a larger database size. However, the species remaining after applying the 5.0% filtering threshold are the same across almost all samples, with approximately the same relative abundances. Therefore, in this case, it appears that most of the species predicted with low relative abundances by larger databases are discarded with the filtering threshold of 5.0%, forming a set of species more similar to the ones predicted by smaller databases.

It should be noted that all taxonomic analysis methods were applied to the same nineteen datasets in this study and, thus, any potential biases in the read numbers of the species affect all methods equally. Notably, if certain methods exhibit a performance benefit due to specific biases (e.g., low or high read coverage of the lowest or highest species in abundance), this would be an interesting observation. However, we have not conducted a detailed analysis of the read coverage of the species identified by each method, as such an analysis was beyond the scope of this work. Consequently, we did not focus on identifying groups of species based on read abundance where each method might perform optimally.

### 3.4 The Comebin/Metabinner Taxonomy Route Provides Useful Insights for Discovering and Annotating Proteins whose Protein Families are Known or Expected

The COMEBin/MetaBinner taxonomy route of *ProteoSeeker* was created as a combination of processes included in the pre‐existing seek mode and the binning methods of COMEBin and MetaBinner. In this route, *ProteoSeeker* employs a custom method for taxonomy assignment by screening each protein against the TPD. If there is at least one hit, the protein is then screened against the TFPD to identify the best hit and its associated taxa. Then, each bin, based on its proteins and their taxonomy classifications, is associated with one or more taxa, and then all genes and proteins of that bin are assigned the same taxa. The nr database was used as the reference protein database for filtering in the evaluations. In addition, initial testing was performed on using the Uniref50 and Uniref90 as protein databases for filtering, as they are of significantly smaller sizes. Due to the reason that many headers of proteins from these Uniref databases include general taxonomy information (e.g., “root”, “bacteria”), the results were not as encouraging as the results acquired by utilizing nr as the protein database to be filtered. *ProteoSeeker*, however, is designed to be able to handle such data and extract the taxonomy information provided in the headers of proteins based on the header‐style of the Uniref databases.

In general, the Kraken2 taxonomy route with the proper filtering threshold(s) performs better in terms of species identification and quantification for a sample compared to the COMEBin/MetaBinner taxonomy route. There are two main reasons for the latter route being a necessary part of the pipeline: the first reason is that the process of binning is a crucial analysis process of analyzing a metagenomic dataset. The taxonomy process itself, as shown by the stage‐specific execution times of *ProteoSeeker*, is a much faster process than binning and does not act as a bottleneck to the pipeline. We believe that for metagenomic datasets including a multitude of microorganisms, whose genomes are yet to be identified and documented, binning the reads may be a more appropriate approach than trying to directly assign species to them primarily based on information originating from known and documented genomes. Binning is partially based on general biological factors, which are used to group contigs coming from the same organisms without including comparisons with known sequences. Hence, each bin may later act as a group of contigs, in turn of reads, genes, and proteins originating from the same organism. The second reason is that the COMEBin/MetaBinner taxonomy route appears to provide insightful results based on the taxonomy classification of proteins from known or expected protein families. This observation regarding the latter taxonomy route can be explained based on the capability of *ProteoSeeker* to base the taxonomy process on protein families associated with the ones to which the proteins of interest are known or expected to belong. More specifically, for each enzyme tested with experimentally confirmed functionality and originating from a sample with an available FASTQ dataset, the taxonomy classification of its best hit (with the lowest E‐value) was obtained by screening it against the nr database of NCBI using the online blastp suite. The latter classification was set as the correct classification for each enzyme and it was compared to the classification provided for the same enzyme by both taxonomy routes of *ProteoSeeker*. It should be emphasized that the COMEBin/MetaBinner taxonomy route is more biased toward making a correct classification compared to the Kraken2 taxonomy route because the COMEBin/MetaBinner taxonomy route utilizes a filtered protein database formed from the nr database which was also used by the blastp suite, while the databases used by the Kraken2 taxonomy route are the Kraken2/Bracken Refseq indexes of the Standard‐8, Standard‐16 and Standard collections. The COMEBin/MetaBinner taxonomy route for each enzyme, via both COMEBin and MetaBinner, except for the case of MetaBinner for CA‐KR1, was able to identify the correct species or genus. According to the results of the Kraken2 taxonomy route, the taxonomy classification of CA‐KR1 was not inferred based on the databases of the Standard‐8 and Standard‐16 collections, and the species and genus predicted based on the database of the Standard collection did not match the target species and genus, respectively. For CA_201 and AL_17, an incorrect species of the right genus was identified for each case of taxonomy route and database. It should be noted that the relative abundances of the species associated with the CA‐KR1, CA_201, and AL_17 enzymes by the Kraken2 taxonomy route are all below 0.1%, while by the COMEBin/MetaBinner taxonomy route are above 0.1%. The misclassification of enzymes by the COMEBin/MetaBinner taxonomy route, while basing its analysis on a filtered nr database, may occur because the protein of the best hit for an enzyme against the non‐filtered nr (based on the blastp suite) has been discarded during filtering. Alternatively, the protein may have no hits against the TPD, or the classification of the bin containing the enzyme may differ from the correct one due to another classification having the highest frequency based on the bin's proteins.

### 3.5 The Kraken2 Taxonomy Route Should be Prioritized Over the Comebin/Metabinner Taxonomy Route for General Taxonomic Classification Purposes and can be Modeled to Excel in Different Evaluation Metrics

We recommend prioritizing the Kraken2 taxonomy route for general taxonomic purposes due to its superior performance in various evaluation metrics. However, the COMEBin/MetaBinner taxonomy route can offer valuable results, particularly for proteins with well‐known protein families. More specifically, applying the COMEBin/MetaBinner taxonomy route, additionally to the Kraken2 taxonomy route, is recommended when analyzing proteins whose families are known or expected, based on filtering the nr database and providing the corresponding family codes as input. Another useful case of applying the latter route is to analyze proteins based on filtering a protein database that contains multiple proteins associated with the proteins of interest by providing associated protein names and family codes (if any) as input to *ProteoSeeker*. While the COMEBin/MetaBinner taxonomy route generally ranks lower than different combinations of Kraken2 databases and filtering thresholds, it remains useful in specific contexts. In addition, Kraken2's flexibility with filtering thresholds offers insights for threshold selection tailored to the sample type. Examining the filtered species based on the different thresholds provided, in general, provides an insight to the user about which threshold is the most suitable one based on the sample being analyzed and using that threshold to re‐bin the contigs in another run. *ProteoSeeker* can easily be configured and run starting by performing gene prediction or binning in the taxonomy mode based on the selected filtering threshold.

As demonstrated in this study, regarding the taxonomy classification, users can prioritize sensitivity, accuracy, precision, F1 score, the Jaccard index, and L1 norm or analyze the taxonomy of proteins from known or expected protein families. This can be achieved by applying either the Kraken2 taxonomy route with a Kraken2/Bracken database and one or more filtering thresholds or the COMEBin/MetaBinner taxonomy route using a protein database, a set of protein family codes, a set of protein names, and a minimum length for the contigs to be binned as input to *ProteoSeeker*. In principle, the COMEBin/MetaBinner taxonomy route scored moderately in relation to the selected best‐scoring Kraken2 combinations of databases and filtering thresholds, for most metrics and samples.

Utilizing the nr database in the COMEBin/MetaBinner taxonomy route is a challenging task based on the aspect of disk space. As part of future work, we are planning to reform the Uniref50 and Uniref90 databases, by including in the headers of their proteins the species documented for those proteins, based on the information available in the UniProtKB/Swiss‐Prot protein database. These reformed protein databases could be tested on whether they could be used as alternative options to the nr database for the type 2 analysis of the seek mode or the COMEBin/MetaBinner taxonomy route of the taxonomy mode of *ProteoSeeker*.

### 3.6 Execution Time Analysis of *Proteoseeker* based on its Taxonomy Mode Evaluation

Total and stage‐specific execution times for *ProteoSeeker* were computed based on the runs of analyzing the nineteen gold standard samples for its taxonomy mode evaluation. The total execution times of *ProteoSeeker* do not include the time spent downloading and converting the SRA datasets to FASTQ, as well as filtering the protein database for the COMEBin/MetaBinner taxonomy route. In addition, the total execution time of *ProteoSeeker* for the species‐abundance categories of 10, 40, 120, 500, and 1000 is at most 61, 43, 121, 141, and 313 min, respectively. There is evident variety regarding the total execution time of *ProteoSeeker* between the different samples of each category. This variety cannot be explained based on the size of the FASTQ files analyzed by *ProteoSeeker* for each sample. It can be attributed mainly to the stage‐specific execution times of the assembly and binning stages. According to the stage‐specific execution times of *ProteoSeeker*, the assembly and binning stages are the most time‐consuming steps and components of the total execution time. However, in samples with ten species, the taxonomy stage also occupies a significant portion of the total execution time, while remaining below 10 min. It should be noted that the stages prior to and including the gene prediction stage are common between the different taxonomy methods of each sample as they were performed once, only for the run based on the Kraken2 taxonomy route with the database of the Standard‐8 collection. In general, we can observe that the time needed for the assembly increases as the number of species in the samples also increases. The binning stage seems to demand approximately the same amount of time as the assembly for the COMEBin/MetaBinner taxonomy route. For the Kraken2 taxonomy route, binning is performed much faster. The binning duration for the COMEBin/MetaBinner taxonomy route is based in part on the minimum contig length specified. In our case, this was the length of 1000 nucleotides. It seems that the higher the number of the contigs satisfying the minimum contig length criterion, the higher the binning time, regardless of the initial size of the sample.

In general, for each sample, the execution time of *ProteoSeeker* increases from the smallest Kraken2 database to the largest, followed by MetaBinner and then COMEBin, both run based on filtered databases from the nr database. One should of course take into consideration the fact that Kraken2 was run without memory mapping and COMEBin was run by utilizing a GPU. In the opposite case for either tool, running Kraken2 with memory mapping or COMEBin without a GPU significantly increases the time of the taxonomy stage and the time for the binning stage, respectively.

The analysis based on the taxonomy evaluation for the total execution time of *ProteoSeeker* in relation to the size and the species‐abundance of the samples showed that the execution time is proportionally dependent on the species‐abundance of the samples of simulated origin.

Time execution analysis was not performed solely for the seek mode of *ProteoSeeker*. The execution time analysis for the taxonomy mode of *ProteoSeeker* was a more valid approach due to the existence of the gold standard samples, which contained simulated reads corresponding to specific species with known relative abundances and biases, allowing for a controlled analysis. The latter analysis offered the ability to make associations between the total and stage‐specific execution times of *ProteoSeeker* with the sizes and species abundance of the samples. In addition, most of the stages of the seek mode are identical to those of the taxonomy mode, e.g., SRA to FASTQ conversion, creation of a profile and a filtered protein database, quality control, preprocessing the reads, assembly, gene prediction and annotation, protein clustering. Therefore, any observations made regarding the execution time analysis of these stages based on the taxonomy mode and its evaluation can also apply to the same stages of the seek mode. Furthermore, we note certain observations we have made throughout the application of *ProteoSeeker* in numerous datasets up to this day, including its runs on datasets related to enzyme discovery projects. These observations are related to both the seek and taxonomy modes of *ProteoSeeker* and focus mainly on the later stages of the seek mode for which no observations were made through the taxonomy mode evaluation. Providing as input numerous protein families or generic protein names to *ProteoSeeker* may lead to the creation of large SPD, TPD, SFPD, and TFPD databases, which will cost additional time regarding the stages that include their screening in both the seek and taxonomy modes. Abbreviations used as protein names should be encompassed by empty spaces (e.g., to use “CA” as the abbreviation for CAs as a protein name it should be provided as “CA”). *ProteoSeeker* can be configured to stop after these databases are created for size and content evaluation by the user. Screening a large SPD or TPD database is much less time‐consuming than screening a large SFPD or TFPD database. The type 1 analysis of the seek mode is based on screening the SPD, while the type 2 analysis of the seek mode is based on screening the SFPD. In addition, the Kraken2 taxonomy route is not affected by the sizes of these databases but the COMEBin/MetaBinner taxonomy route is, specifically by the TPD and TFPD databases.

Screening the Pfam database in the seek mode is relatively fast except if the number of proteins reaching that stage is exceptionally large. Using CD‐HIT to cluster proteins, identify representatives, and reduce their total number without losing valuable information is easy to apply and time‐efficient. *ProteoSeeker* can directly run after the gene prediction stage, starting with gene annotation and CD‐HIT application. Therefore, CD‐HIT can play a key role in controlling the number of proteins and reducing their redundancy in subsequent stages (e.g., stages that include screening the SPD, TPD, SFPD, and TFPD databases), thus decreasing their execution times. Additionally, protein family identification based on the best hits from screening proteins against the UniProtKB/Swiss‐Prot database, input motif screening, and topology predictions made by Phobius are typically rapid processes. These steps do not act as bottlenecks for *ProteoSeeker*.

## 4 Conclusion

In response to the increased complexity and resource demands of existing metagenomic data analysis pipelines, we developed *ProteoSeeker*, a comprehensive and precise analytical tool. *ProteoSeeker* processes metagenomic (e.g., environmental and other metagenomes), genomic (genomes or contigs), and proteomic data (protein sequences in FASTA format). It identifies proteins and screens them based on user‐defined protein families while performing taxonomic analysis. Designed for non‐expert users, *ProteoSeeker* integrates state‐of‐the‐art tools and automates workflows with minimal user input. By enhancing accessibility and consistency across studies, *ProteoSeeker* surpasses the capabilities of individual integrated tools to identify proteins of interest. It allows users to easily modify the behavior or type of tools and databases utilized in its pipeline. *ProteoSeeker* has already been successfully used to discover novel enzymes for industrial applications, including a highly promising CA biocatalyst for biomimetic carbon capture. These analyses and discoveries support the efficacy of *ProteoSeeker's* seek mode and its adaptable analysis types, facilitating protein discovery and annotation related to specific protein families.

*ProteoSeeker*’s evaluations underscore the effectiveness of Kraken2's taxonomy route for taxonomy classification. The COMEBin/MetaBinner taxonomy route may prove insightful for analyzing proteins from specific protein families when relevant codes and protein names are provided. Users can focus on various evaluation metrics (true positives, false positives, sensitivity, accuracy, etc.) by using the appropriate databases and filtering thresholds in the Kraken2 taxonomy route, allowing for tailored analyses. With the introduction of *ProteoSeeker* to the biotechnology community, we expect more widespread use of metagenomic analyses. This comprehensive tool is expected to accelerate the discovery of novel biocatalysts and biomolecules and drive biotechnological innovation forward by leveraging the abundance of publicly available (meta)genomic data. By emphasizing accessibility and automation, *ProteoSeeker* is set to become an essential resource for bioscientists and biotechnologists, enhancing innovation in protein discovery and biotechnological research across various sectors.

## 5 Experimental Section

### Dataset Dependencies in *ProteoSeeker*

The functions of *ProteoSeeker* are dependent on the existence of certain datasets. The user can update these datasets, at any time, by utilizing the instructions and dedicated scripts provided in *ProteoSeeker's* GitHub repository, requiring only the UniProtKB/Swiss‐Prot flat file or the Pfam HMM database as input. The Swiss‐Prot/UniProtKB‐related datasets have been the result of performing an analysis on the UniProtKB/Swiss‐Prot flat file (downloaded on 04/08/2023). The flat file of UniProtKB/Swiss‐Prot includes information about the reviewed proteins of the UniProtKB/Swiss‐Prot protein database. Based on the analysis of the flat file the length, the organism, the name(s), the Pfam profile(s), and the protein family assigned to each protein were collected. Each protein family was associated with a group of proteins. Each protein was associated with a set of Pfam profiles where each profile has a frequency. Two sets of profiles are considered to be identical if their profiles and their frequencies match. The frequency for each set of profiles of the proteins in a protein family was computed. Consequently, each protein family, through its protein group, was associated with one or more sets of profiles of maximum frequency within the protein group, a mean and median length, and a set of protein names. Each protein name was also accompanied by a frequency. Two sets of profiles, from two different proteins, are identical when each profile of one set is of the same type and has the same frequency as another profile of the other set and vice versa. The different sets of profiles that may have been associated with a protein family at this point may include profiles of the same type. The Pfam‐related datasets have been the result of performing an analysis on the Pfam HMM database (downloaded at 29/05/2024). These datasets contain a correspondence between Pfam accession numbers, Pfam short names, and Pfam profile lengths.

### Overview of *ProteoSeeker's* Functionalities, Data Preparation, and Input Processing

*ProteoSeeker* offers two primary and independent functionalities. The first functionality is termed “seek functionality” and it is applied through the “seek mode” of *ProteoSeeker* (Figure 2). This mode includes three types of analysis, termed “type 1”, “type 2” and “type 3” respectively. Type 1 analysis includes searching for putative proteins that contain domains corresponding to the profiles associated with the selected protein families. Type 2 analysis includes searching for putative proteins that have at least one hit of low enough E‐value against the “seek filtered protein database” (SFPD). Type 3 analysis includes both type 1 and type 2 analysis. The second functionality is termed “taxonomy functionality” and it is applied through the “taxonomy mode” of *ProteoSeeker* (Figure 3). It includes two distinct taxonomy routes, each of which performs a taxonomic analysis of the putative proteins and binning of the contigs. The first route, the “Kraken2 (taxonomy) route,” performs taxonomy classification of the reads using Kraken2, estimates abundances with Bracken, and then bins the contigs based on the taxonomy classification of their reads. The second route, the “COMEBin/MetaBinner (taxonomy) route”, at first bins the contigs, then isolates the proteins which include at least one domain corresponding to a profile from the TPD, runs DIAMOND to screen the latter proteins against the TFPD, determines the taxon or taxa associated with the best hit of each protein and eventually assigns one or more taxa to a bin and by extension to its genes and proteins.

All stages of the pipeline implemented by *ProteoSeeker* will be described based on the application of its processes for a type 3 analysis (both type 1 and type 2 analyses) of the seek mode and both taxonomy routes of the taxonomy mode.

Based on the datasets provided from the analysis of the UniProtKB/Swiss‐Prot database, each protein family selected corresponds to one or more sets of Pfam profiles based on which in turn a unique set of profiles is formed. The latter set is used by HMMER to create a database of profiles.^[^ ^58^, ^59^ ^]^ Selecting protein families for the seek or the taxonomy mode leads to the creation of a “seek profile database” (SPD) or a “taxonomy profile database” (TPD), respectively. *ProteoSeeker* may also accept as input a profile database directly. When selecting protein families, the following stage is to utilize a protein database. The protein database selected as the most suitable one for the analyses performed by *ProteoSeeker* is the non‐redundant (nr) protein database of NCBI. The protein database is filtered based on the protein names associated with the selected protein families. The protein names of each selected protein family are filtered based on a threshold. This threshold is a percentage and computes the value of the minimum frequency required for a protein name not to be discarded, based on the maximum frequency of the protein names. Each protein name that surpasses the threshold is used in filtering the protein database and creating either the “seek filtered protein database” (SFPD) or the “taxonomy filtered protein database” (TFPD). This filtering is carried out by a supportive Python command‐line tool and module, which can take as input sets of protein names and corresponding file names and can run on multiple processes in parallel  (more information about this tool can be found in Supporting Text: 1.2, Supporting Information). The SFPD or TFPD is converted to a database by DIAMOND. The user may provide a file with protein sequences in FASTA format and its corresponding database directly. Also, the user can provide his own set of protein names and select whether only the names he provided will be used to create the SFPD or TFPD, or whether the names he provided will be added (if not present already) to the ones associated with the selected protein families. Utilizing a protein database is mandatory when the type 2 analysis of the seek mode is to be performed or when taxonomic analysis is to be carried out through the COMEBin/MetaBinner taxonomy route. All processes related to utilizing the selected protein families for the taxonomy mode (e.g., creating the TPD, filtering the protein database, and creating the TFPD) are omitted when the taxonomic analysis is based on the Kraken2 taxonomy route. Using Kraken2 comes with the need for indexes which come in a variety of composition and size and can be downloaded as pre‐built databases.

One mandatory input for *ProteoSeeker* is an SRA code (RUN accession) from the SRA database of NCBI or a dataset (file input). The user‐specified input file(s) may be single‐end or paired‐end reads in FASTQ format, assembled reads (contigs) or genomes or protein sequences in FASTA format. Based on each case of file input, specific initial stages of the pipeline implemented in *ProteoSeeker* are omitted. Hence, *ProteoSeeker* can process W(M)GS data and genomic and proteomic datasets. *ProteoSeeker's* pipeline includes the SRA Toolkit (sra‐tools).^[^ ^60^, ^61^ ^]^ If an SRA code is provided, then *ProteoSeeker* utilizes the SRA Toolkit to automatically download the SRA file associated with the code, validate its integrity, and convert it to its corresponding compressed FASTQ files. These FASTQ files are then processed by the next stages of *ProteoSeeker's* pipeline as if the user had provided these files directly as input to *ProteoSeeker*.

### Seek Mode and Taxonomy Mode

Both modes of *ProteoSeeker* begin with running FastQC to perform several quality checks (quality control) of the reads.^[^ ^62^ ^]^ A brief analysis of the results from FastQC follows which is targeted at the number of the reads and the overrepresented sequences identified. The next stage of the analysis is preprocessing the reads with BBDuk from BBMap^[^ ^63^ ^]^ which involves filtering the reads based on quality and length analyses and trimming the reads based on a list of sequences (“adapters file”) (for more information about these analyses see Supporting Text: 1.6, Supporting Information). A user can provide a file that contains adapter sequences or use an existing one that contains adapters generally used by various NGS platforms. The user can also select whether the overrepresented sequences identified by FastQC will be added to the adapters file. At this point, FastQC is run again to analyze the preprocessed reads. Assembly is the next crucial stage of *ProteoSeeker*. It is performed by MEGAHIT and provides contigs as part of the output.^[^ ^64^ ^]^

The following processes are solely part of the taxonomy mode. The COMEBin/MetaBinner taxonomy route at this stage uses the binning tool of COMEBin or MetaBinner to bin the contigs. Binning is based on the contigs and the preprocessed FASTQ files. The selection of the binning tool was based on a number of criteria. These include the capability to bin contigs based on their reads, running through the command line, an installation process with as few requirements as possible and without demanding large in‐size database(s), the capability to utilize multiple CPUs, few input requirements to run and a straightforward output regarding the bins of the input contigs. These criteria resulted in the selection of COMEBin and MetaBinner, as other tools we examined did not satisfy one or more of these criteria. Both COMEBin and MetaBinner were selected because either is more suitable for different cases of input data and do not follow the same binning methodology. COMEBin in addition can utilize a GPU which can offer a significant advantage.

The Kraken2 taxonomy route utilizes Kraken2 with a Kraken2 database to predict species by analyzing the reads of the sample and associate these species with the reads. Next, Bracken, based on a Bracken database, estimates the abundances and relative abundances of the Kraken2 predicted species, at a specific taxonomy rank (e.g., class, order, family, genus). While Bracken performs this estimation, it can be tuned to discard species predicted by Kraken2 with abundances (number of reads assigned to the species) below a certain threshold. At this stage, *ProteoSeeker* optionally applies user‐defined thresholds (“‐kt/–kraken‐threshold” option) to filter species provided as output by Bracken. These thresholds are not applied to taxonomy ranks other than species. A filtering threshold may target the absolute or relative abundances of the species. A threshold can be an integer (and applies to absolute abundances) or a float (and applies to relative abundances). In addition, the user can select the automatic computation of the filtering threshold via the Shannon index based on the results of Bracken, for “non‐gut” or “gut” samples, as described by the method and formula proposed by Poussin et al.^[^ ^44^ ^]^ The Shannon index is computed by KrakenTools. Species with absolute or relative abundance below the threshold are excluded from further processing, although their associated reads are not reassigned. One of the filtering thresholds (which can be user‐defined) is used by *ProteoSeeker* to select the corresponding set of filtered species to be used by subsequent stages of the analysis (e.g., binning). The additional thresholds produce secondary, filtered species lists for alternative or comparative analyses.

For clarity, the following describes the processing of the contigs as implemented in the Kraken2 taxonomy route, which occurs after mapping the reads to the contigs. If a set of reads, which solely belong to one or more contigs, are not associated with any species, then those contigs will also not be associated with any species. Lack of association with at least one species includes the case where the reads have only been associated with higher taxonomic ranks (e.g., genus). In cases where a contig is equally associated with multiple species with the same highest frequency of reads, the contig will not be associated with any species, will not undergo further processing, and will not be taken into account in the taxonomy‐based binning.

The next step of *ProteoSeeker*, for both its modes, is the application of the gene prediction tool, FragGeneScanRs.^[^ ^65^ ^]^ This tool can identify protein‐coding regions, including prokaryotic genes, and provide their protein sequences. Moreover, translation tables based on NCBI's genetic codes are provided for utilization. Information is also collected for each gene regarding the presence or not of start and stop codons, their length, and their distance from the ends of their contig. CD‐HIT is then applied to perform clustering of the protein sequences.^[^ ^66^, ^67^ ^]^ CD‐HIT can handle extremely large databases and can help to reduce the computational demands of subsequent stages by reducing the redundancy of the proteins.

The following steps are part solely of the seek mode of *ProteoSeeker*. *ProteoSeeker* proceeds, in type 1 analysis, into screening the proteins against the SPD with HMMER. The proteins subject to further annotation, contain each protein that scored at least one hit against the SPD (protein set 1). Additionally, in type 2 analysis, the rest of the proteins (not part of protein set 1) are screened against the SFPD by DIAMOND. Those that scored at least one hit with an E‐value equal to or below a specific threshold (by default 1e‐70), are also subject to further annotation (protein set 2). Both protein sets are then combined and subjected to screening, through HMMER, against all the profiles of the Pfam database. Furthermore, protein set 1 is screened against the SFPD by DIAMOND. Both protein sets are also screened by DIAMOND against the UniProtKB/Swiss‐Prot protein database.

The following steps are part solely of the taxonomy mode of *ProteoSeeker*. At this point, a common stage between both taxonomy routes is that of read mapping. Reads are mapped to contigs through Bowtie2.^[^ ^68^, ^69^ ^]^ For the COMEBin/MetaBinner taxonomy route each bin and its taxa are both quantified based on the reads mapped to the contigs of the bin. The relative abundance of a bin and of its taxa is the proportion of the reads mapped to the contigs of the bin against the total number of preprocessed reads. For the same taxonomy route, *ProteoSeeker* performs screening of the proteins against the TPD, through HMMER. Proteins with at least one hit are then screened through DIAMOND against the TFPD. The TFPD database is parsed, and every protein of the TFPD is associated with one or more taxa based on the information in its header. In turn, each putative protein is associated with one or more taxa based on its hit (if any) of the lowest E‐value against the TFPD. The TaxIds of the latter taxa and their lineages are found based on TaxonKit.^[^ ^70^ ^]^ Output related to the taxonomic analysis at this point is formulated with the help of csvtk.^[^ ^71^ ^]^ Each contig contains one or more genes and thus is associated with their proteins. In turn, each bin contains several contigs and is associated with their proteins. By using proteins that were assigned at least one taxon and based on the proteins included in a bin, each bin is assigned one or more taxa accompanied by frequencies. The frequency of each taxon is the number of times this taxon is found associated with the proteins of the bin. The taxon or taxa with the highest frequency for the bin are identified and assigned to that bin. The same taxon or taxa are also assigned to every gene and protein of the bin. Therefore, in the case of the COMEBin/MetaBinner taxonomy route, a bin may be associated with taxa at various ranks beyond species, such as strain, subspecies, or genus. If no taxa were associated with a bin, then that bin, its contigs, genes, and proteins do not receive taxonomic annotation. In the Kraken2 taxonomy route, by combining the information from the filtered (non‐discarded) species (if any) assigned to the reads, and from the read mapping to the contigs by Bowtie2, species are associated with the contigs. Each species associated with a contig is accompanied by a frequency, equal to the number of reads associated with that species and mapped to that contig. If a single species for a contig has the highest frequency, then that species is assigned to the contig, otherwise no species is assigned to the contig. Based on the species of the contigs, the latter are binned. Hence, each bin of contigs is directly assigned a single species and also quantified based on the quantification provided for the same species. The next step is the assignment of the species of a bin to each gene and protein of that bin.

### Additional Analyses, Annotation, and Output Generation

In the case of applying solely the taxonomy mode of *ProteoSeeker* the pipeline does not perform any other analysis after this stage. Otherwise, the pipeline goes on to perform more analyses in the seek mode. The first analysis is to predict the transmembrane topology of the proteins of both protein sets, by Phobius.^[^ ^72^ ^]^ Phobius is a tool that performs transmembrane topology and signal peptide prediction in proteins. The second analysis is to search for user‐input motifs in the protein. The third analysis is to predict the protein family of each putative protein. This is accomplished by determining the hit with the lowest E‐value acquired by screening the putative protein against the UniProtKB/Swiss‐Prot database through DIAMOND and identifying the protein family of that hit. The mean and median length of the latter family are determined based on *ProteoSeeker's* dataset dependencies, which were provided from the analysis of the UniProtKB/Swiss‐Prot database. Moreover, the length of the putative protein is compared with the mean length of the predicted protein family and their difference is documented in the results and final annotation.

Lastly, for both the seek and taxonomy modes, annotation files are generated by *ProteoSeeker*. These files contain a synopsis of the information collected for each putative protein from either or both of the two modes. A summary of the type of results found in the annotation files can be found in Table S1 (Supporting Information). The user can specify which information is to be included in the annotation files based on the seek mode, the taxonomy mode or both modes. In addition, if *ProteoSeeker* is run after or up to a specific stage in the pipeline for a sample or dataset already analyzed previously, it is able to collect the available information from a previous run, combine them, and output the previous and new information in a new set of annotation files, supposing that the proper output name is set (based on the previous run). This is possible as at each stage of the pipeline *ProteoSeeker* stores the information collected for the putative proteins in specific files which it can be set to utilize in future runs of the same sample or dataset. It should be noted that, at each possible termination point of *ProteoSeeker*, execution time information for the completed stages of the pipeline, as well as for the entire run, is provided.

### Availability of Data and Materials

The datasets, source code of *ProteoSeeker version 1.0.0*, the versions of the tools included in *ProteoSeeker's* pipeline version 1.0.0 and the collection dates of the databases which support the evaluation and conclusions of this article are publicly available in the GitHub repository of *ProteoSeeker* with the tag “v1.0.0” at “https://github.com/SkretasLab/ProteoSeeker” ^[^ ^28^ ^]^ and with the version 1.0.0 used in the manuscript deposited in the DOI‐assigning repository Zenodo at “https://doi.org/10.5281/zenodo.13944968”.^[^ ^73^ ^]^ *ProteoSeeker* is also shipped as a docker image through Docker Hub by the repository “skretaslab/proteoseeker” at “https://hub.docker.com/r/skretaslab/proteoseeker”.^[^ ^29^ ^]^ To access the website of *ProteoSeeker* please visit “www.skretaslab.gr/proteoseeker”.^[^ ^30^ ^]^ The datasets of the gold standard samples used in the evaluation and the methodology of computing the filtering threshold applied to the species after Bracken's application based on the Shannon index are described in the work of Poussin et al.^[^ ^44^ ^]^ The datasets of the gold standard samples used in the evaluation are also described in the Supporting Information (Supporting Text: 1.4). The SRA accession numbers of the gold standard samples and other related information are provided in Table S3 (Supporting Information).

### Project name: *ProteoSeeker*

Project home page: https://github.com/SkretasLab/ProteoSeeker.^[^ ^28^ ^]^ and www.skretaslab.gr/proteoseeker.^[^ ^30^ ^]^ Archived version: https://doi.org/10.5281/zenodo.13944968.^[^ ^73^ ^]^ Operating system(s): The Linux operating system is needed to install and run the command‐line tool. The Linux operating system with the amd64 architecture and a platform that supports running Docker images is needed to run the Docker image.

### Programming Language: Python, Bash

Other requirements: The Anaconda^[^ ^74^ ^]^ environment is needed for the command‐line tool of *ProteoSeeker*. A system capable of deploying Docker containers from Docker images is needed for the Docker image of *ProteoSeeker*. License: GNU General Public License (GPLv3) for the source code of *ProteoSeeker*.

## Conflict of Interest

The authors declare that they have no conflict of interest.

## Author Contributions

G.S. and D.Z. designed and managed the project. G.F. wrote the source code of *ProteoSeeker*, and the code used for the evaluations of *ProteoSeeker*. G.F. and D.B. wrote the documentation for *ProteoSeeker*. G.F. and D.B. performed the evaluations of the seek and taxonomy modes of *ProteoSeeker*. K.R. performed experimental analyses of CAs and ALs. DN performed experimental analyses of ALs. P.S. performed experimental analyses of CAs. G.F., D.B., K.R., D.N., D.Z., and G.S. wrote the manuscript. D.Z. and G.S. revised the manuscript. All authors wrote and approved the final manuscript.

## Supporting information



Supporting Information

Supplemental Table

## Data Availability

The data that support the findings of this study are openly available in SkretasLab/ProteoSeeker: ProteoSeeker v1.0.0 at https://doi.org/10.5281/zenodo.13944968, reference number 13944968.
