# Supplementary material for: ProteoSeeker: A Feature‐Rich Metagenomic Analysis Tool for Accessible and Comprehensive Metagenomic Exploration
Source: Adv Sci (Weinh). 2025 Mar 25;12(19):2414877. doi: 10.1002/advs.202414877 (PMC12097006; doi:10.1002/advs.202414877)
Supplement: Supplementary file 1 — Supporting Information [file ADVS-12-2414877-s001.docx]

Supporting Information

*ProteoSeeker:* A Feature-Rich Metagenomic Analysis Tool for

Accessible and Comprehensive Metagenomic Exploration

*Georgios Filis, Dimitra Bezantakou, Konstantinos Rigkos, Despina Noti, Pavlos Saridis, Dimitra Zarafeta*, Georgios Skretas**

[*skretas@fleming.gr](mailto:*skretas@fleming.gr)

*[zarafeta@fleming.gr](mailto:zarafeta@fleming.gr)

# 1. Supporting Text

## 1.1 Tools versions

All the tools are automatically installed by the installation process of *ProteoSeeker* or have already been set in the Docker image of *ProteoSeeker*. *ProteoSeeker* version 1.0.0 (“v.1.0.0”) was used for the evaluation of the taxonomy mode of *ProteoSeeker*. For some packages more than one installation method is provided. If the first method is not successful then the next one is attempted. The version of Anaconda used for the installation and working environment of *ProteoSeeker*, the tools and their versions included in the pipeline of *ProteoSeeker* version 1.0.0 are the following:

1. Anaconda: 24.1.2
2. BBMap: 39.01
3. Bowtie2: 2.5.3
4. CD-HIT: 4.8.1
5. COMEBin:
   1. From conda: 1.0.4 - Used for the evaluation.
   2. From GitHub: Branch: "1.0.4".
6. DIAMOND: 2.1.9
7. FastQC 0.12.1
8. HMMER: 3.4
9. Kraken2:
   1. From conda: 2.1.3 - Used for the evaluation.
   2. From GitHub: Branch: "v2.1.3"
10. Bracken:
    1. From conda: 2.9 - Used for the evaluation.
    2. From GitHub: Branch: "v2.9"
11. MEGAHIT: 1.2.9
12. MetaBinner:
    1. From GitHub: Branch: "master", Hash: "50a1281e8200d705a744736f23efe53c6048bbe8" - Used for the evaluation.
    2. From conda: 1.4.4
13. SRA Toolkit (sra-tools): 3.1.0
14. TaxonKit: 0.16.0
15. csvtk: 0.30.0
16. FragGeneScanRs: 1.1.0

## 1.2 Filtering a protein database

The process of filtering a protein database according to certain protein names is based on a Python command-line tool and module which was developed specifically for this process. This tool takes as input a set of file names and lists of protein names. Each file eventually will contain the proteins of the protein database which contain in their headers at least one of the protein names of its corresponding list of protein names. This process has been parallelized. The protein database is divided into chunks. Each chunk is processed individually. The filtering takes place simultaneously for each chunk and is based on its list of protein names. This module is utilized by *ProteoSeeker* to filter the protein database when the type 2 analysis of the seek mode or the COMEBin/MetaBinner taxonomy route of the taxonomy mode is applied, and the filtered protein database has not already been generated in a previous run.

## 1.3 Execution time of *ProteoSeeker* for samples associated with discovered enzymes experimentally verified

Execution times based on the runs of *ProteoSeeker* version 1.0.0 for the taxonomy mode evaluation for proteins of known or expected protein families are provided in this section. These runs were performed in an Ubuntu 24.04 LTS system with 124 GBs of RAM and 32 CPUs available. More specifically, during the evaluation the seek mode and either the COMEBin/MetaBinner or the Kraken2 taxonomy route of the taxonomy mode were utilized in each run. The results of this evaluation are shown in Table S7 of this file (Supporting Information). The samples analyzed are DRR163688, SRR3961740 and SRR17771278 which respectively led to the discovery of CA_KR1, CA_201 and AL_17. The pair of compressed FASTQ files for the same SRA samples have the respective sizes of 893.6 MB, 2.1 GB and 12 GB. A run of *ProteoSeeker* was performed separately to acquire and process each SRA sample. The execution time of collecting and processing each SRA sample has been added to the total execution time of the analysis for the same sample. In general, the stage of filtering the protein database is not mandatory when the goal is centered at discovering proteins or/and annotating them rapidly without applying the type 2 analysis of the seek mode or the COMEBin/MetaBinner taxonomy route for taxonomic analysis. In this case, pre-existing filtered protein databases were utilized by *ProteoSeeker.* The end of the CD-HIT stage can be considered the end of protein discovery as the representatives of the protein clusters have been identified and are considered as the putative proteins to be further processed and annotated. All execution times mentioned below are based on the COMEBin/MetaBinner taxonomy route through the application of COMEBin as this is one of the most time-consuming taxonomic analysis methods provided by *ProteoSeeker*, typically being closely approximated by the same taxonomy route through the application of MetaBinner. Hence, it is a valid choice to use as the basis for an execution time analysis of *ProteoSeeker’s* pipeline, regarding the application of both its seek and taxonomy modes in the same run. The time elapsed for *ProteoSeeker* to analyze samples DRR163688, SRR3961740 and SRR17771278 up to the CD-HIT stage without accounting for the protein database filtering stage is 7.72 min, 57.29 min and 182.24 min respectively. Similarly, the total execution time elapsed for the pipeline to finish for the same samples is 11.86 min, 34.16 min and 267.78 min respectively. It should be noted that the runs utilizing the Kraken2 taxonomy route are faster in terms of the total execution time of the pipeline.

## 1.4 “Gold standard” samples

The 19 “gold standard” samples used to evaluate *ProteoSeeker’s* taxonomy mode and execution time were originally created in the work of Poussin *et al.^[1]^* and are publicly available as paired-end FASTQ files​ with raw sequencing data, in the National Center for Biotechnology Information (NCBI) repository under NCBI BioProject ID PRJNA669653.^[2]^ The samples were either simulated (15 samples) or “real” (4 samples) from a known microbial composition standard.

The methodology for creating the fifteen synthetic datasets involved generating sequencing reads using the ART simulation tool. The authors relied on a snapshot of the NCBI bacterial taxonomy (dated mid-2017) and 1886 complete bacterial genomes to define the pool of potential species. The simulation parameters were configured to mimic next-generation sequencing (NGS) reads from an Illumina HiSeq4000 sequencer, with 2 × 150-bp paired-end reads. Samples with varying complexity were created, including those with low and medium complexity (fewer than 500 species) and higher complexity (500 or more species) and included unbiased sets, alongside those intentionally biased toward AT- or GC-rich genomes (>60% AT/GC) (detailed list on Table **S3**). ​To simulate host contamination, reads from mouse cecal samples were mapped onto the mouse genome and added as contaminants, representing 8-11% of the total reads. The species for each sample were drawn randomly from a list of candidate species found in mouse gut microbiomes, with the number of reads corresponding to species abundance. For higher complexity samples, species coverage followed a log-normal distribution based on CAMI guidelines and included closely related species to evaluate the capability of computational analysis pipelines to discriminate bacteria at the species level. For low and medium complexity samples, species were randomly selected from a list of candidate species identified from mouse gut microbiomes. ​

In addition to the simulated datasets, four real metagenomics sequencing samples were generated using the commercially available ZymoBIOMICS™ Microbial Community DNA Standards, which corresponded to mixtures of genomic DNA extracted from pure cultures of eight bacteria and two yeasts at known ratios. ​ These samples were sequenced in a 2 × 151-bp paired-end run on an Illumina HiSeq4000 sequencer. ​

This methodology ensured a diverse and carefully controlled set of benchmark datasets representative of various microbiome compositions and complexities, enabling a thorough evaluation of the taxonomic profiling methods of the *ProteoSeeker* pipeline, expressed as relative abundances.

## 1.5 Metrics used in the evaluation of the taxonomy mode

The metrics used in the evaluation of the Kraken2 and COMEBin/MetaBinner taxonomy routes in the taxonomy mode are the following:

1. True Positive (TP) hits:

The number of common species between the predicted species by *ProteoSeeker* and the species which belong to the gold standard sample (“gold standard species”).

1. False Positive (FP) hits:

The number of species predicted by *ProteoSeeker* which species do not belong to the gold standard species.

1. False Negative (FN) hits:

The number of gold standard species that were not predicted by *ProteoSeeker*.

1. Sensitivity:


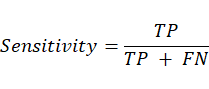


1. Precision:


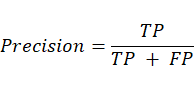


1. Accuracy:


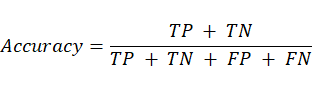


1. F1 Score


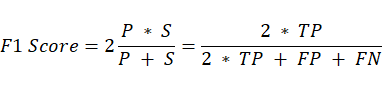


1. Jaccard Index

A: Set of gold standard species.

B: Set of predicted species by *ProteoSeeker*.


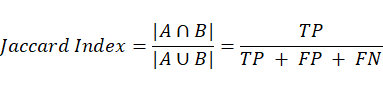


1. L1 norm


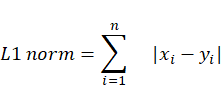


where n is the number of species present in the set of gold standard species,
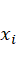
 is the relative abundance of the i^th^ species in the predicted profile, and
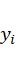
 is the relative abundance of the i^th^ species in the gold standard profile. The relative abundance of each predicted species, based on the COMEBin/MetaBinner taxonomy route, is rounded up to two decimal places. The relative abundance of each predicted species, based on the Kraken2 taxonomy route, is converted to a percentage and is rounded up to three decimal places based on the output of Bracken. A species that is not present in the predicted species gets a relative abundance equal to 0%.

## 1.6 Preprocessing the reads - Quality and length analyses by BBDuk

BBDuk performs quality and length analyses in the pipeline of *ProteoSeeker* as an early stage of the pipeline for preprocessing the reads. The values provided to the options of “bbduk.sh” in *ProteoSeeker* version 1.0.0 are not modifiable, except for the maximum number of CPUs and amount of RAM allowed for BBDuk to utilize, based on “-t/--threads” and “-umr/--bbduk-max-ram” options respectively. Subsequent versions of *ProteoSeeker* will make more options of BBDuk available for modification. Currently, the command for running BBDuk in *ProteoSeeker’s* pipeline is based on the following options and their values, excluding the ones associated with file input and output:

● interleaved=f

● ktrim=r

● k=21

● mink=11

● hammingdistance=2

● qtrim=rl

● trimq=20

● minavgquality=20

● minlength=25

● tpe=t

● tbo=t

● threads=Specified by the “-t/--threads” option of *ProteoSeeker*.

● -XmxYg: “Y” is specified by the “-umr/--bbduk-max-ram” option of *ProteoSeeker*.

# 2. Supporting Figures


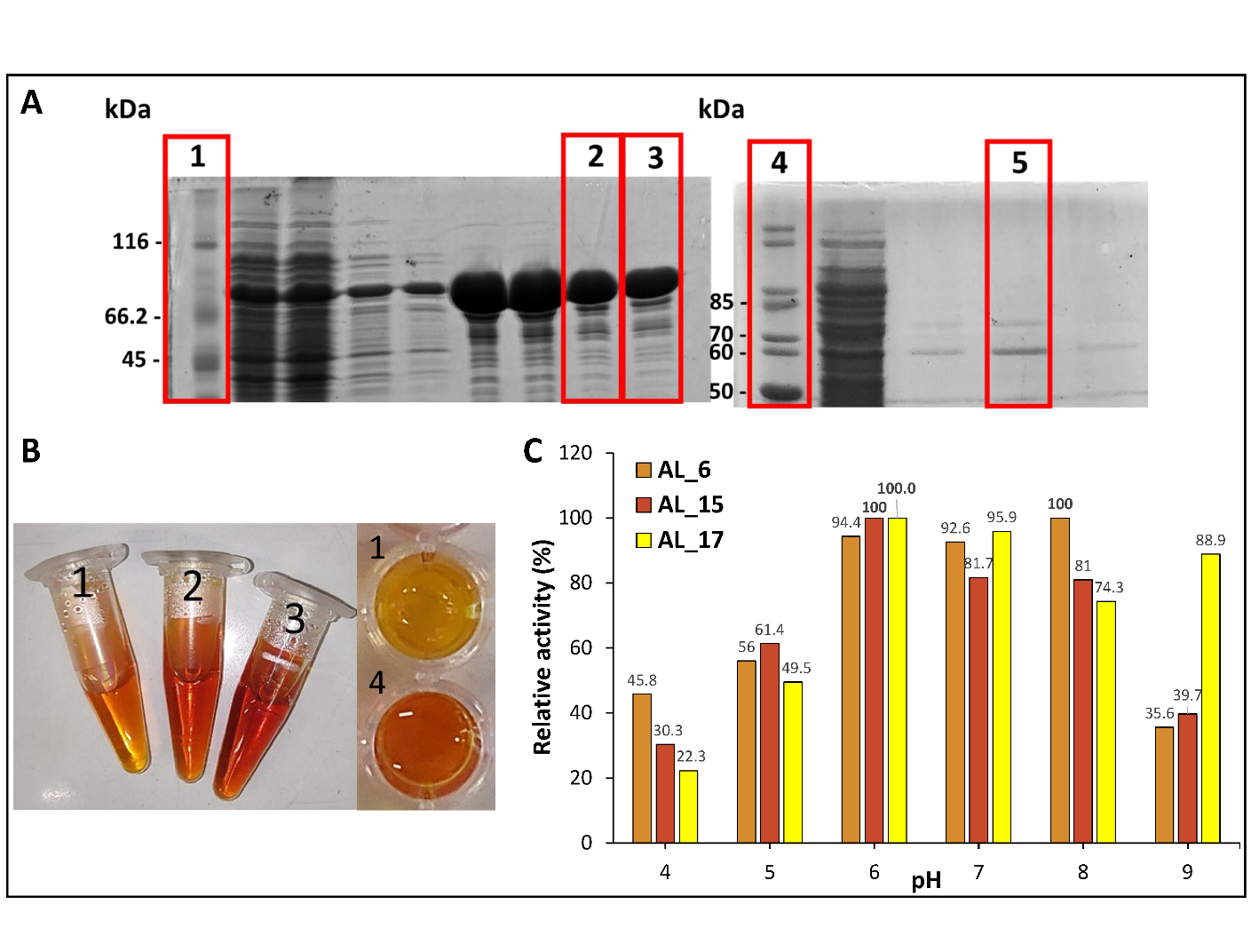


**Figure S1. Biochemical characterization of IMAC purified AL_6, AL_15 and AML_17.** **(A)** SDS-PAGE analysis of AL_6 (Lane 2), AL_15 (Lane 3) and AL_17 (Lane 5) amylases on a 15% polyacrylamide gel, with a protein standard in Lane 1 and Lane 4. **(B)** DNS assay of the amylases at 70 °C for 1 hour (0.05% w/v final starch concentration).^[3]^ Samples include: No enzyme reaction (1), AL_6 (2), AL_15 (3), and AL_17 (4). During the amylolytic reaction, the reducing sugars produced react with 3,5-Dinitrosalicylic acid (DNS), forming 3-Aminosalicylic acid, which generates a distinct red color. The intensity of the red color indicates amylase activity. **(C)** pH-dependent activity of AL_6, AL_15 and AL_17. Samples were incubated in buffers with pH ranging from 4 to 9, followed by DNS assay (0.05% w/v final starch concentration) to measure enzymatic activity via absorbance at 540 nm. The maximum absorbance value of all enzymes was set as 100% relative activity, and all other values were normalized to these maxima.


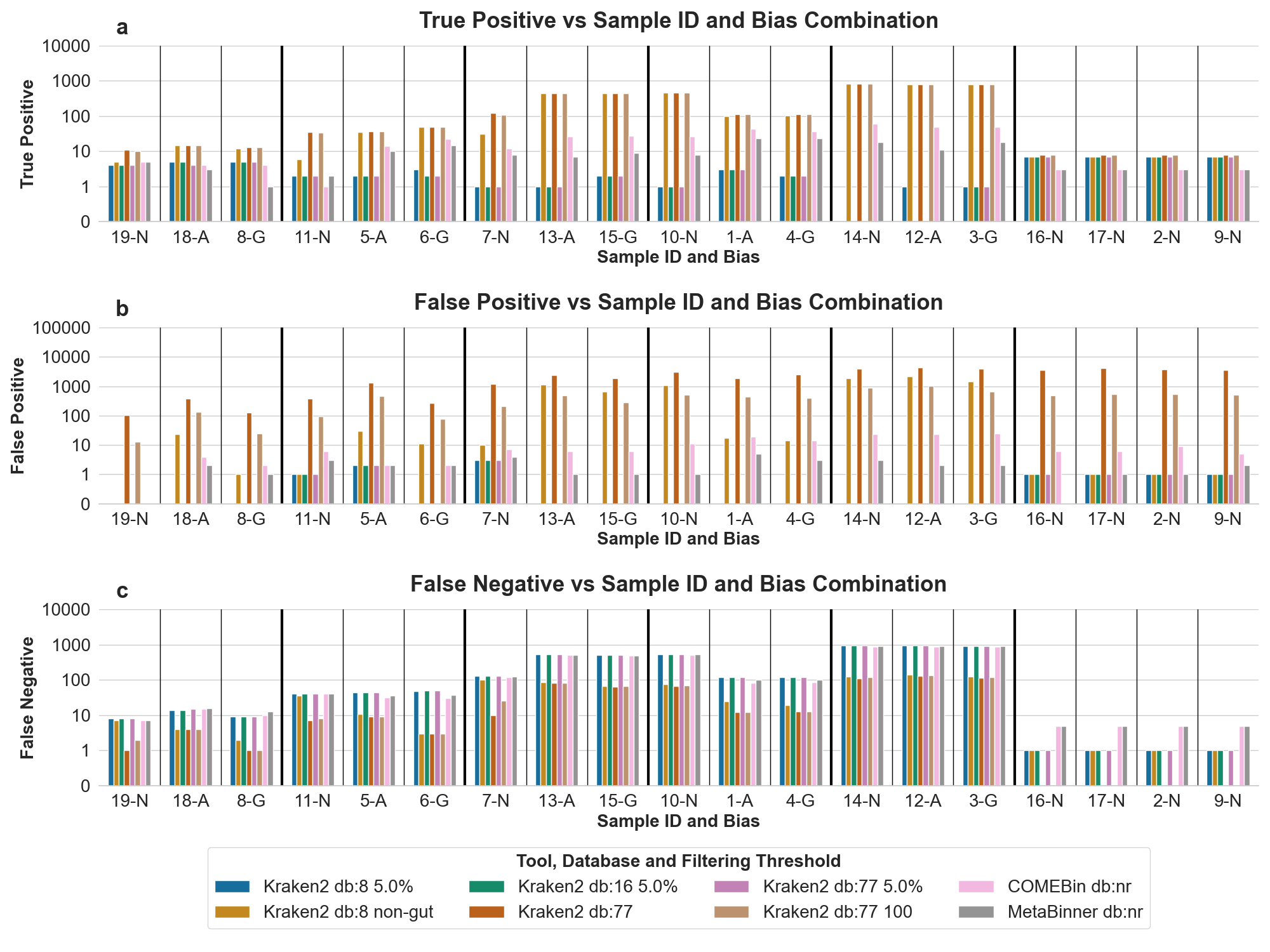


**Figure S2.** True positive **(a)**, false positive **(b)** and false negative **(c)** hits of the taxonomy mode evaluation results with the samples sorted based on their species-abundances and biases. The results were acquired by the *ProteoSeeker* runs for the selected combinations of Kraken2 databases and filtering thresholds plus the COMEBin/MetaBinner taxonomy methods for each sample of the 19 gold standard datasets. The selected combinations regarding the Kraken2 taxonomy route include the database of the Standard-8 collection with the filtering threshold of 5.0% (“Kraken2 db:8 5.0%”) and the filtering threshold computed based on non-gut samples (“Kraken2 db:8 non-gut”), the database of the Standard-16 collection with the filtering threshold of 5.0% (“Kraken2 db:16 5.0%”) and the database of the Standard collection without a filtering threshold (“Kraken2 db:77”) and with the filtering thresholds of 5.0% (“Kraken2 db:77 5.0%”) and of 100 (“Kraken2 db:77 100”). The COMEBin/MetaBinner taxonomy route was applied through COMEBin with the non-redundant (nr) protein database as the filtering target (“COMEBin db:nr”) and through MetaBinner with the nr protein database as the filtering target (“MetaBinner db:nr”). The samples are sorted into groups of species-abundance. Samples 19, 18, 8 for 10 species from simulated reads, samples 11, 5, 6 for 40 species, samples 7, 13, 15 for 120 species, samples 10, 1, 4 for 500 species, samples 14, 12, 3 for 1000 species and samples 16, 17, 2, 9 for 10 species from cultures. The letters “N”, “A” and “G” on the labels stand for “No bias”, “AT-rich bias” and “GC-rich bias” respectively. The y-axis is presented on a logarithmic scale (base 10), with an adjustment to include zero values.


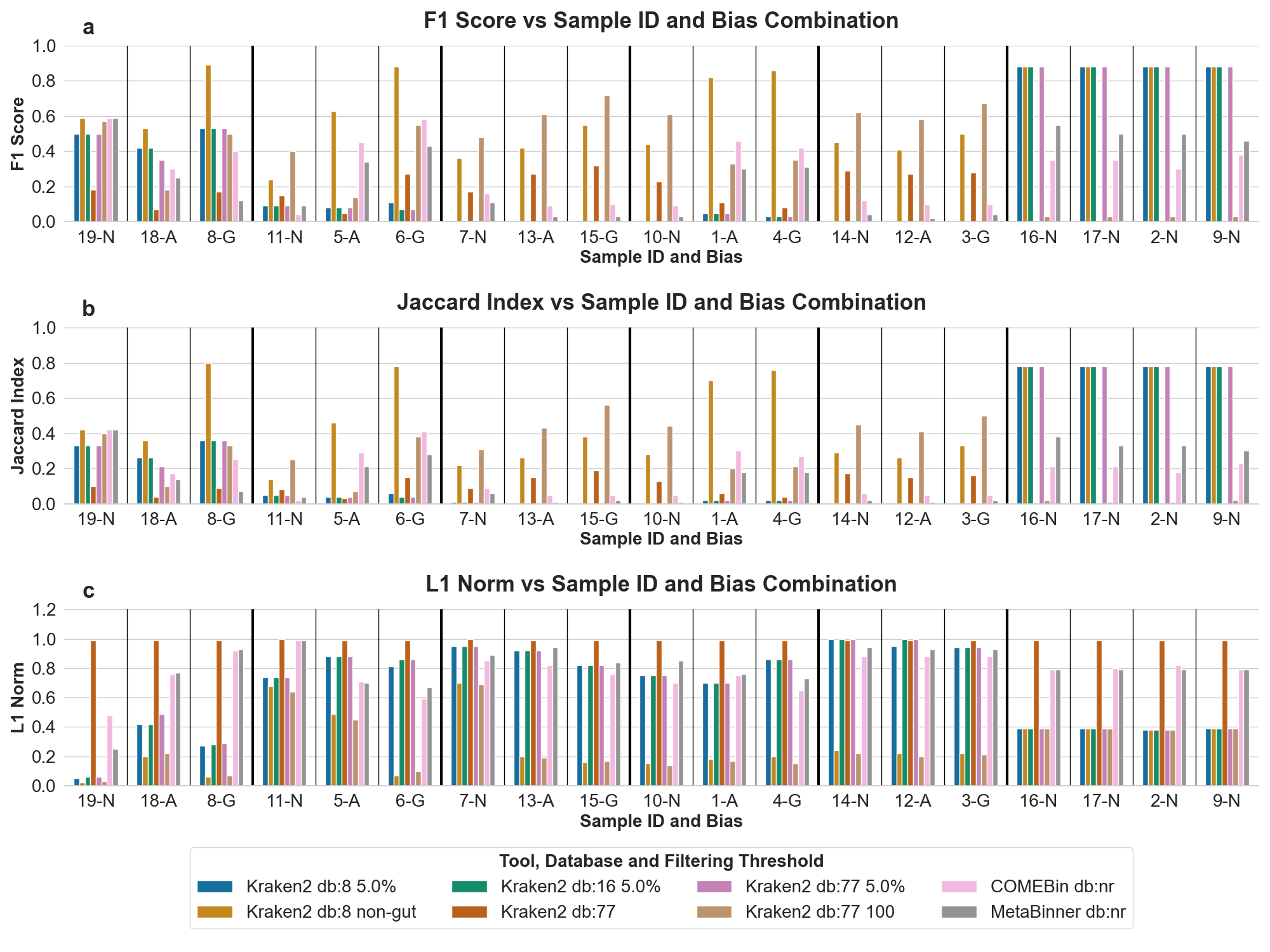


**Figure S3.** F1 score **(a)**, Jaccard index **(b)** and L1 norm **(c)** of the taxonomy mode evaluation results with the samples sorted based on their species-abundances and biases. The results were acquired by the *ProteoSeeker* runs for the selected combinations of Kraken2 databases and filtering thresholds plus the COMEBin/MetaBinner taxonomy methods for each sample of the 19 gold standard datasets. The selected combinations regarding the Kraken2 taxonomy route include the database of the Standard-8 collection with the filtering threshold of 5.0% (“Kraken2 db:8 5.0%”) and the filtering threshold computed based on non-gut samples (“Kraken2 db:8 non-gut”), the database of the Standard-16 collection with the filtering threshold of 5.0% (“Kraken2 db:16 5.0%”) and the database of the Standard collection without a filtering threshold (“Kraken2 db:77”) and with the filtering thresholds of 5.0% (“Kraken2 db:77 5.0%”) and of 100 (“Kraken2 db:77 100”). The COMEBin/MetaBinner taxonomy route was applied through COMEBin with the non-redundant (nr) protein database as the filtering target (“COMEBin db:nr”) and through MetaBinner with the nr protein database as the filtering target (“MetaBinner db:nr”). The samples are sorted into groups of species-abundance. Samples 19, 18, 8 for 10 species from simulated reads, samples 11, 5, 6 for 40 species, samples 7, 13, 15 for 120 species, samples 10, 1, 4 for 500 species, samples 14, 12, 3 for 1000 species and samples 16, 17, 2, 9 for 10 species from cultures. The letters “N”, “A” and “G” on the labels stand for “No bias”, “AT-rich bias” and “GC-rich bias” respectively.


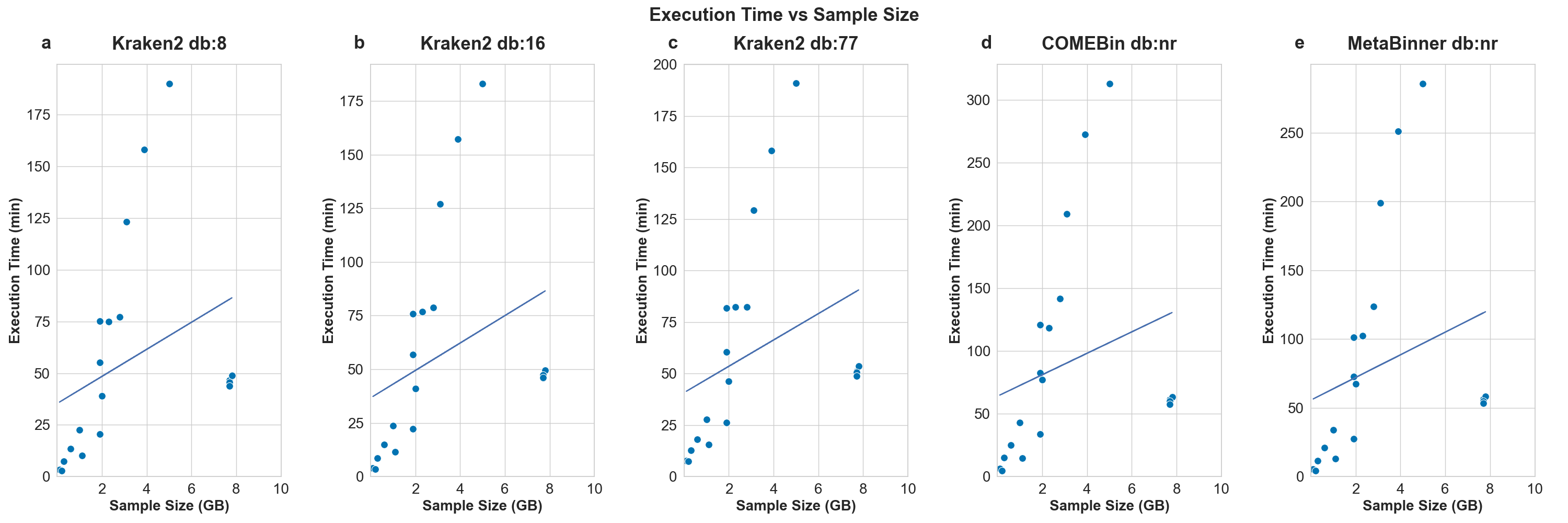


**Figure S4.** The total execution time of *ProteoSeeker*, based on its taxonomy mode evaluation, for each database and in turn for each of the 19 gold standard datasets, relative to their sizes. The databases used in the evaluation, for the Kraken2 taxonomy route are **(a)** the Standard-8 collection, **(b)** the Standard-16 collection, **(c)** the Standard collection and for the COMEBin/MetaBinner taxonomy route, **(d)** for COMEBin and for **(e)** MetaBinner the non-redundant (nr) protein database of NCBI. The size of a sample is computed in GB, which equals the sum of the sizes of the paired-end FASTQ files extracted from the SRA dataset of the sample. In the case of the Kraken2 taxonomy route, the effect of the filtering threshold is considered negligible as it may affect the number of species to be considered during the binning process and subsequent stages of the pipeline which effects in total account for very small changes in the execution time. The total execution time for each of the datasets does not include the time needed to download and process the SRA sample by *ProteoSeeker*. The initial stages of the pipeline, up to the stage of gene prediction, are common and have the exact same execution time for the different runs of the same sample. A straight line has been fitted to the data points of each group of runs based on the combination of the taxonomy route and database.


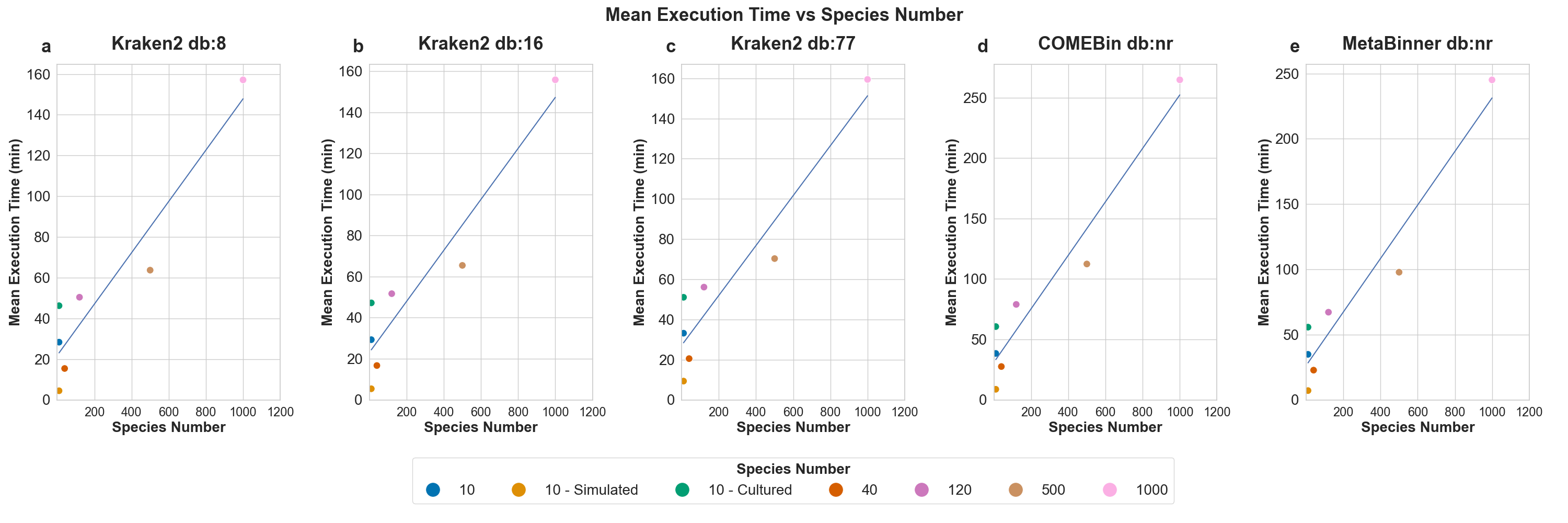


**Figure S5.** The mean execution time of *ProteoSeeker*, based on its taxonomy mode evaluation, for each database and in turn for subgroups of the 19 gold standard datasets, relative to their species abundance. The databases used in the evaluation, for the Kraken2 taxonomy route are **(a)** the Standard-8 collection, **(b)** the Standard-16 collection, **(c)** the Standard collection and for the COMEBin/MetaBinner taxonomy route, **(d)** for COMEBin and for **(e)** MetaBinner the non-redundant (nr) protein database of NCBI. In the case of the Kraken2 taxonomy route, the effect of the filtering threshold is considered negligible as it may affect the number of species to be considered during the binning process and subsequent stages of the pipeline which effects in total account for very small changes in the execution time. For the group of 10 species the mean execution time has also been computed for the two subgroups of 10 species based on whether they originate from simulated reads or from cultures. Each of the total execution times, based on which the mean times were computed, does not include the time needed to download and process the SRA sample by *ProteoSeeker*. The initial stages of the pipeline, up to the stage of gene prediction, are common and have the exact same execution time for the different runs of the same sample. A straight line has been fitted to the data points of each group of runs based on the combination of the taxonomy route and database.

# Supporting Tables

**Table S1**. A summary of the results documented in the annotation files generated from a *ProteoSeeker* run after applying both the seek and taxonomy modes. The term “Frequency” in this case is defined as the number of times the category of information may be associated with a protein.

| **Mode** | **Category** | **Field** | **Description** | **Frequency** |
| --- | --- | --- | --- | --- |
| seek/taxonomy | protein | id | The protein ID of the protein. | once |
| seek/taxonomy | protein | sequence | The amino acid sequence of the protein. | once |
| seek/taxonomy | protein | length | The length of the protein, in residues. | once |
| seek | domain | description | The description of the domain as provided in the output of HMMER. | any |
| seek | domain | target name | The target name of the domain as provided in the output of HMMER. | any |
| seek | domain | accession | The accession of the domain as provided in the output of HMMER. | any |
| seek | domain | e-value | The E-value of the overall sequence/profile comparison (including all domains), of the domain, as provided in the output of HMMER. | any |
| seek | domain | bitscore | The Bit score of the overall sequence/profile comparison (including all domains), inclusive of a null2 bias composition correction to the score, of the domain, as provided in the output of HMMER. | any |
| seek | domain | c-evalue | The c-Evalue ("conditional E-value") of the domain as provided in the output of HMMER. | any |
| seek | domain | i-evalue | The i-Evalue ("independent E-value") of the domain as provided in the output of HMMER. | any |
| seek | domain | profile | The start and end of the MEA alignment of the domain with respect to the profile, numbered according to the residues of the profile, as provided in the output of HMMER. | once |
| seek | domain | envelope | The start and end of the domain envelope on the protein sequence, numbered according to the residues of the protein sequence, as provided in the output of HMMER. | once |
| taxonomy | bin | bin id | The ID of the bin to which the protein is assigned. The protein belongs to the bin which includes the contig, in which contig the gene of the protein was found. | once |
| taxonomy | taxonomy | taxonomy origin | Information about the taxon(a) associated with the protein, if any. It indicates whether the taxonomy was determined by the Kraken2 taxonomy route ("K") or the COMEBin/MetaBinner taxonomy route utilizing COMEBin ("C") or the COMEBin/MetaBinner taxonomy route utilizing MetaBinner ("M"). For the COMEBin/MetaBinner taxonomy route it also indicates whether the taxonomy of the protein was determined directly or indirectly. The first case refers to identifying one or more taxa associated with the protein based on its hit(s) against the taxonomy filtered protein database. The second case refers to assigning one or more taxa to the protein based on the respective taxa assigned to its bin. | once |
| seek | topology | transmembrane topology | The start and end of the region predicted as a signal peptide or a transmembrane region on the protein sequence, numbered according to the residues of the protein sequence, accompanied by more specialized information about the nature of the transmembrane region or of parts of the signal peptide, as provided in the output of Phobius. | any |
| seek | Swiss-Prot | accession number | The accession number of the hit with the lowest E-value acquired by the results from screening the protein against the UniProtKB/Swiss-Prot database through DIAMOND. | once |
| seek | Swiss-Prot | e-value | The E-value of the hit from UniProtKB/Swiss-Prot. | once |
| seek | Swiss-Prot | identity | The Identity percentage of the hit from UniProtKB/Swiss-Prot. | once |
| seek | Swiss-Prot | bitscore | The Bit score of the hit from UniProtKB/Swiss-Prot. | once |
| seek | Swiss-Prot | protein family | The protein family of the hit from UniProtKB/Swiss-Prot. The protein family is based on information from the UniProtKB/Swiss-Prot protein database. | once |
| seek | Swiss-Prot | mean length | The mean length of the protein family of the hit from UniProtKB/Swiss-Prot, in residues. | once |
| seek | Swiss-Prot | median length | The median length of the protein family of the hit from UniProtKB/Swiss-Prot, in residues. | once |
| seek | Swiss-Prot | length difference | The difference of the protein length with the mean length of the protein family of the hit from UniProtKB/Swiss-Prot, in residues. | once |
| seek | Swiss-Prot | length relative change | The relative change of the protein length with the mean length of the protein family of the hit from UniProtKB/Swiss-Prot, in residues. | once |
| seek | Swiss-Prot | family difference | If no protein families had been initially selected in the "seek" mode, then the label "-" is given in this field. On the contrary, if any of the selected protein families provided in the "seek" mode matches the predicted one, then the label "1" is given to the field. If no such a match is found, then the label "0" is given to the field. | once |
| seek | seek filtered protein database | ID | The ID of the hit with the lowest E-value acquired by the results from screening the protein against the "seek filtered protein database" (sfpd) through DIAMOND. | once |
| seek | seek filtered protein database | e-value | The E-value of the hit described above (for the ID). | once |
| seek | seek filtered protein database | identity | The Identity percentage of the hit described above (for the ID). | once |
| seek | seek filtered protein database | bitscore | The Bit score percentage of the hit described above (for the ID). | once |
| seek | motif | motif sequence | The sequence of the motif identified in the protein. | any |
| seek | motif | motif region | The start and end of the motif on the protein sequence, numbered according to the residues of the protein sequence. | any |
| seek/taxonomy | gene | ID | The ID of the gene of the protein, as provided in the output of FragGeneScanRs. | once |
| seek/taxonomy | gene | sequence | The nucleotide sequence of the gene of the protein, as provided in the output of FragGeneScanRs. | once |
| seek/taxonomy | gene | first and last codon | For the first and last codon of the gene sequence of the protein a label "YES" is provided if they match any of the start and end codons widely associated with prokaryotic organisms, respectively. Otherwise, a label "NO" is provided. | once |
| seek/taxonomy | gene | contig edge distance | The distance of the gene from the edges of its contig is provided. Specifically, the distance from the start of the contig until the start of the gene and the distance from the end of the gene until the end of the contig are provided. | once |
| seek/taxonomy | custom | user-defined | Any custom field and its corresponding information provided by the user are noted at the end of the entry for the protein. | once |

**Table S3.** Information about each of the 19 gold standard datasets. For each dataset its sample ID, its SRA code, its species number, its category, its bias and the size of the paired-end FASTQ files extracted from the SRA file and analyzed by *ProteoSeeker* are provided.

| **Sample ID** | **SRA code** | **Species number** | **Category** | **Bias** | **Size (GB)** |
| --- | --- | --- | --- | --- | --- |
| 8 | SRR12829159 | 19 | 10 | GC-rich bias | 0.1297 |
| 19 | SRR12829170 | 12 | 10 | No bias | 0.2049 |
| 18 | SRR12829169 | 14 | 10 | AT-rich bias | 0.3474 |
| 17 | SRR12829162 | NA | 10 | No bias | 7.7 |
| 16 | SRR12829163 | NA | 10 | No bias | 7.7 |
| 9 | SRR12829160 | NA | 10 | No bias | 7.8 |
| 2 | SRR12829161 | NA | 10 | No bias | 7.7 |
| 6 | SRR12829156 | 46 | 40 | GC-rich bias | 0.6313 |
| 5 | SRR12829157 | 52 | 40 | AT-rich bias | 0.9808 |
| 11 | SRR12829158 | 42 | 40 | No bias | 1.1 |
| 7 | SRR12829155 | 132 | 120 | No bias | 1.9 |
| 13 | SRR12829154 | 124 | 120 | AT-rich bias | 1.9 |
| 15 | SRR12829153 | 124 | 120 | GC-rich bias | 1.9 |
| 1 | SRR12829168 | 508 | 500 | AT-rich bias | 2.0 |
| 10 | SRR12829152 | 536 | 500 | No bias | 2.3 |
| 4 | SRR12829167 | 528 | 500 | GC-rich bias | 2.8 |
| 3 | SRR12829164 | 934 | 1000 | GC-rich bias | 3.1 |
| 14 | SRR12829166 | 949 | 1000 | No bias | 3.9 |
| 12 | SRR12829165 | 912 | 1000 | AT-rich bias | 5.0 |

**Table S4.** The Shannon index and the values of the filtering thresholds computed automatically for non-gut and gut samples for each Kraken2 database and each sample of the gold standard dataset.

| Database | Sample | Shannon Index | non-gut filtering value (%) | gut filtering value (%) |
| --- | --- | --- | --- | --- |
| 8 | 1 | 4.06 | 0.1% | 0.0% |
| 8 | 2 | 2.19 | 1.0% | 0.1% |
| 8 | 3 | 5.9 | 0.0% | 0.0% |
| 8 | 4 | 4.34 | 0.1% | 0.0% |
| 8 | 5 | 3.65 | 0.1% | 0.0% |
| 8 | 6 | 3.69 | 0.1% | 0.0% |
| 8 | 7 | 2.6 | 0.1% | 0.0% |
| 8 | 8 | 2.21 | 1.0% | 0.1% |
| 8 | 9 | 2.19 | 1.0% | 0.1% |
| 8 | 10 | 4.64 | 0.0% | 0.0% |
| 8 | 11 | 1.39 | 1.0% | 0.1% |
| 8 | 12 | 5.52 | 0.0% | 0.0% |
| 8 | 13 | 5.35 | 0.0% | 0.0% |
| 8 | 14 | 6.05 | 0.0% | 0.0% |
| 8 | 15 | 4.75 | 0.0% | 0.0% |
| 8 | 16 | 2.17 | 1.0% | 0.1% |
| 8 | 17 | 2.19 | 1.0% | 0.1% |
| 8 | 18 | 2.77 | 0.1% | 0.0% |
| 8 | 19 | 1.42 | 1.0% | 0.1% |
| 16 | 1 | 4.08 | 0.1% | 0.0% |
| 16 | 2 | 2.23 | 1.0% | 0.1% |
| 16 | 3 | 5.94 | 0.0% | 0.0% |
| 16 | 4 | 4.39 | 0.1% | 0.0% |
| 16 | 5 | 3.76 | 0.1% | 0.0% |
| 16 | 6 | 3.72 | 0.1% | 0.0% |
| 16 | 7 | 2.63 | 0.1% | 0.0% |
| 16 | 8 | 2.24 | 1.0% | 0.1% |
| 16 | 9 | 2.22 | 1.0% | 0.1% |
| 16 | 10 | 4.67 | 0.0% | 0.0% |
| 16 | 11 | 1.42 | 1.0% | 0.1% |
| 16 | 12 | 5.55 | 0.0% | 0.0% |
| 16 | 13 | 5.38 | 0.0% | 0.0% |
| 16 | 14 | 6.08 | 0.0% | 0.0% |
| 16 | 15 | 4.78 | 0.0% | 0.0% |
| 16 | 16 | 2.2 | 1.0% | 0.1% |
| 16 | 17 | 2.22 | 1.0% | 0.1% |
| 16 | 18 | 2.85 | 0.1% | 0.0% |
| 16 | 19 | 1.44 | 1.0% | 0.1% |
| 77 | 1 | 4.17 | 0.1% | 0.0% |
| 77 | 2 | 2.33 | 1.0% | 0.1% |
| 77 | 3 | 6 | 0.0% | 0.0% |
| 77 | 4 | 4.48 | 0.1% | 0.0% |
| 77 | 5 | 3.8 | 0.1% | 0.0% |
| 77 | 6 | 3.76 | 0.1% | 0.0% |
| 77 | 7 | 2.74 | 0.1% | 0.0% |
| 77 | 8 | 2.31 | 1.0% | 0.1% |
| 77 | 9 | 2.32 | 1.0% | 0.1% |
| 77 | 10 | 4.77 | 0.0% | 0.0% |
| 77 | 11 | 1.48 | 1.0% | 0.1% |
| 77 | 12 | 5.64 | 0.0% | 0.0% |
| 77 | 13 | 5.44 | 0.0% | 0.0% |
| 77 | 14 | 6.14 | 0.0% | 0.0% |
| 77 | 15 | 4.83 | 0.0% | 0.0% |
| 77 | 16 | 2.29 | 1.0% | 0.1% |
| 77 | 17 | 2.32 | 1.0% | 0.1% |
| 77 | 18 | 2.96 | 0.1% | 0.0% |
| 77 | 19 | 1.47 | 1.0% | 0.1% |

**Table S5.** Information about the different evaluation cases of the taxonomy mode and taxonomy routes of *ProteoSeeker*. For each case, the tools utilized by *ProteoSeeker* in the taxonomy route, and the database utilized in the evaluation are provided. Each gold standard sample was run through each of these 5 combinations of taxonomy route, tools and database.

| **Taxonomy route** | **Tools** | **Database** |
| --- | --- | --- |
| Kraken2 | Kraken2, Bracken, KrakenTools, Bowtie2 | Kraken 2 / Bracken Refseq index, Collection: Standard-8 |
| Kraken2 | Kraken2, Bracken, KrakenTools, Bowtie2 | Kraken 2 / Bracken Refseq index, Collection: Standard-16 |
| Kraken2 | Kraken2, Bracken, KrakenTools, Bowtie2 | Kraken 2 / Bracken Refseq index, Collection: Standard |
| COMEBin/MetaBinner | COMEBin, Bowtie2, HMMER, DIAMOND, TaxonKit, csvtk | NCBI, non-redundant (nr) protein database |
| COMEBin/MetaBinner | MetaBinner, Bowtie2, HMMER, DIAMOND, TaxonKit, csvtk | NCBI, non-redundant (nr) protein database |

**Table S6.** The frequency of the best-scoring combinations for each metric based on all 19 samples of the gold standard dataset.

| **Metric** | **Combination** | **Frequency** |
| --- | --- | --- |
| Accuracy | Kraken2 db:8 non-gut | 7 |
| Accuracy | Kraken2 db:8 1.0% | 6 |
| Accuracy | Kraken2 db:77 non-gut | 5 |
| Accuracy | Kraken2 db:16 1.0% | 5 |
| Accuracy | Kraken2 db:77 5.0% | 4 |
| Accuracy | Kraken2 db:8 5.0% | 4 |
| Accuracy | Kraken2 db:16 non-gut | 4 |
| Accuracy | Kraken2 db:16 5.0% | 4 |
| Accuracy | Kraken2 db:77 1.0% | 3 |
| Accuracy | Kraken2 db:8 500 | 3 |
| Accuracy | Kraken2 db:8 1000 | 3 |
| Accuracy | Kraken2 db:8 0.1% | 2 |
| Accuracy | Kraken2 db:77 0.1% | 2 |
| Accuracy | Kraken2 db:8 0.01% | 2 |
| Accuracy | Kraken2 db:8 100 | 1 |
| Accuracy | Kraken2 db:16 1000 | 1 |
| Accuracy | Kraken2 db:77 500 | 1 |
| Accuracy | Kraken2 db:16 500 | 1 |
| F1 Score | Kraken2 db:8 non-gut | 7 |
| F1 Score | Kraken2 db:16 non-gut | 6 |
| F1 Score | Kraken2 db:77 non-gut | 6 |
| F1 Score | Kraken2 db:8 1.0% | 6 |
| F1 Score | Kraken2 db:16 1.0% | 5 |
| F1 Score | Kraken2 db:8 500 | 5 |
| F1 Score | Kraken2 db:8 1000 | 5 |
| F1 Score | Kraken2 db:77 5.0% | 4 |
| F1 Score | Kraken2 db:8 5.0% | 4 |
| F1 Score | Kraken2 db:16 5.0% | 4 |
| F1 Score | Kraken2 db:8 0.01% | 4 |
| F1 Score | Kraken2 db:77 0.1% | 3 |
| F1 Score | Kraken2 db:77 1.0% | 3 |
| F1 Score | Kraken2 db:16 500 | 3 |
| F1 Score | Kraken2 db:8 0.1% | 2 |
| F1 Score | Kraken2 db:16 0.1% | 2 |
| F1 Score | Kraken2 db:16 1000 | 2 |
| F1 Score | Kraken2 db:16 0.01% | 2 |
| F1 Score | Kraken2 db:8 100 | 1 |
| F1 Score | Kraken2 db:77 500 | 1 |
| False Negative (Abundance of Species Unique to the Gold Standard Group) | Kraken2 db:77 | 19 |
| False Negative (Abundance of Species Unique to the Gold Standard Group) | Kraken2 db:77 gut | 13 |
| False Negative (Abundance of Species Unique to the Gold Standard Group) | Kraken2 db:77 100 | 10 |
| False Negative (Abundance of Species Unique to the Gold Standard Group) | Kraken2 db:77 500 | 9 |
| False Negative (Abundance of Species Unique to the Gold Standard Group) | Kraken2 db:16 | 9 |
| False Negative (Abundance of Species Unique to the Gold Standard Group) | Kraken2 db:77 1000 | 8 |
| False Negative (Abundance of Species Unique to the Gold Standard Group) | Kraken2 db:77 0.01% | 8 |
| False Negative (Abundance of Species Unique to the Gold Standard Group) | Kraken2 db:8 | 8 |
| False Negative (Abundance of Species Unique to the Gold Standard Group) | Kraken2 db:77 non-gut | 8 |
| False Negative (Abundance of Species Unique to the Gold Standard Group) | Kraken2 db:16 1000 | 7 |
| False Negative (Abundance of Species Unique to the Gold Standard Group) | Kraken2 db:16 500 | 7 |
| False Negative (Abundance of Species Unique to the Gold Standard Group) | Kraken2 db:8 0.01% | 7 |
| False Negative (Abundance of Species Unique to the Gold Standard Group) | Kraken2 db:16 0.01% | 7 |
| False Negative (Abundance of Species Unique to the Gold Standard Group) | Kraken2 db:8 1000 | 7 |
| False Negative (Abundance of Species Unique to the Gold Standard Group) | Kraken2 db:8 500 | 7 |
| False Negative (Abundance of Species Unique to the Gold Standard Group) | Kraken2 db:8 100 | 7 |
| False Negative (Abundance of Species Unique to the Gold Standard Group) | Kraken2 db:16 100 | 7 |
| False Negative (Abundance of Species Unique to the Gold Standard Group) | Kraken2 db:8 gut | 4 |
| False Negative (Abundance of Species Unique to the Gold Standard Group) | Kraken2 db:8 0.1% | 4 |
| False Negative (Abundance of Species Unique to the Gold Standard Group) | Kraken2 db:16 gut | 4 |
| False Negative (Abundance of Species Unique to the Gold Standard Group) | Kraken2 db:77 0.1% | 3 |
| False Negative (Abundance of Species Unique to the Gold Standard Group) | Kraken2 db:16 non-gut | 3 |
| False Negative (Abundance of Species Unique to the Gold Standard Group) | Kraken2 db:16 0.1% | 3 |
| False Negative (Abundance of Species Unique to the Gold Standard Group) | Kraken2 db:8 non-gut | 2 |
| False Positive (Abundance of Species Unique to the Predicted Group) | Kraken2 db:16 5.0% | 19 |
| False Positive (Abundance of Species Unique to the Predicted Group) | Kraken2 db:77 5.0% | 19 |
| False Positive (Abundance of Species Unique to the Predicted Group) | Kraken2 db:8 5.0% | 19 |
| False Positive (Abundance of Species Unique to the Predicted Group) | Kraken2 db:8 1.0% | 10 |
| False Positive (Abundance of Species Unique to the Predicted Group) | Kraken2 db:16 1.0% | 9 |
| False Positive (Abundance of Species Unique to the Predicted Group) | Kraken2 db:8 non-gut | 6 |
| False Positive (Abundance of Species Unique to the Predicted Group) | Kraken2 db:16 non-gut | 5 |
| False Positive (Abundance of Species Unique to the Predicted Group) | Kraken2 db:77 1.0% | 5 |
| False Positive (Abundance of Species Unique to the Predicted Group) | Kraken2 db:77 non-gut | 3 |
| Jaccard Index | Kraken2 db:8 non-gut | 7 |
| Jaccard Index | Kraken2 db:8 1.0% | 6 |
| Jaccard Index | Kraken2 db:77 non-gut | 5 |
| Jaccard Index | Kraken2 db:16 non-gut | 5 |
| Jaccard Index | Kraken2 db:16 1.0% | 5 |
| Jaccard Index | Kraken2 db:8 500 | 5 |
| Jaccard Index | Kraken2 db:8 1000 | 5 |
| Jaccard Index | Kraken2 db:77 5.0% | 4 |
| Jaccard Index | Kraken2 db:8 5.0% | 4 |
| Jaccard Index | Kraken2 db:16 5.0% | 4 |
| Jaccard Index | Kraken2 db:8 0.01% | 4 |
| Jaccard Index | Kraken2 db:77 1.0% | 3 |
| Jaccard Index | Kraken2 db:16 500 | 3 |
| Jaccard Index | Kraken2 db:8 0.1% | 2 |
| Jaccard Index | Kraken2 db:77 0.1% | 2 |
| Jaccard Index | Kraken2 db:16 0.01% | 2 |
| Jaccard Index | Kraken2 db:16 0.1% | 1 |
| Jaccard Index | Kraken2 db:8 100 | 1 |
| Jaccard Index | Kraken2 db:16 1000 | 1 |
| Jaccard Index | Kraken2 db:77 500 | 1 |
| L1 Norm | Kraken2 db:77 100 | 14 |
| L1 Norm | Kraken2 db:77 1000 | 13 |
| L1 Norm | Kraken2 db:77 500 | 13 |
| L1 Norm | Kraken2 db:16 100 | 13 |
| L1 Norm | Kraken2 db:16 500 | 13 |
| L1 Norm | Kraken2 db:77 gut | 13 |
| L1 Norm | Kraken2 db:77 0.01% | 12 |
| L1 Norm | Kraken2 db:16 gut | 12 |
| L1 Norm | Kraken2 db:16 1000 | 11 |
| L1 Norm | Kraken2 db:77 non-gut | 11 |
| L1 Norm | Kraken2 db:16 0.01% | 10 |
| L1 Norm | Kraken2 db:16 non-gut | 10 |
| L1 Norm | Kraken2 db:8 0.01% | 9 |
| L1 Norm | Kraken2 db:8 1000 | 9 |
| L1 Norm | Kraken2 db:8 500 | 9 |
| L1 Norm | Kraken2 db:8 100 | 9 |
| L1 Norm | Kraken2 db:8 gut | 8 |
| L1 Norm | Kraken2 db:16 0.1% | 7 |
| L1 Norm | Kraken2 db:8 non-gut | 7 |
| L1 Norm | Kraken2 db:8 0.1% | 7 |
| L1 Norm | Kraken2 db:77 0.1% | 6 |
| L1 Norm | Kraken2 db:16 1.0% | 5 |
| L1 Norm | Kraken2 db:8 1.0% | 5 |
| L1 Norm | Kraken2 db:77 5.0% | 4 |
| L1 Norm | Kraken2 db:77 1.0% | 4 |
| L1 Norm | Kraken2 db:16 5.0% | 4 |
| L1 Norm | Kraken2 db:8 5.0% | 4 |
| Precision | Kraken2 db:8 5.0% | 15 |
| Precision | Kraken2 db:77 5.0% | 14 |
| Precision | Kraken2 db:16 5.0% | 14 |
| Precision | Kraken2 db:8 1.0% | 11 |
| Precision | Kraken2 db:16 1.0% | 10 |
| Precision | Kraken2 db:16 non-gut | 6 |
| Precision | Kraken2 db:8 non-gut | 6 |
| Precision | Kraken2 db:77 1.0% | 5 |
| Precision | Kraken2 db:77 non-gut | 3 |
| Precision | Kraken2 db:16 0.1% | 1 |
| Precision | Kraken2 db:8 0.1% | 1 |
| Sensitivity | Kraken2 db:77 | 19 |
| Sensitivity | Kraken2 db:77 gut | 13 |
| Sensitivity | Kraken2 db:77 100 | 10 |
| Sensitivity | Kraken2 db:77 500 | 9 |
| Sensitivity | Kraken2 db:16 | 9 |
| Sensitivity | Kraken2 db:8 | 8 |
| Sensitivity | Kraken2 db:77 1000 | 8 |
| Sensitivity | Kraken2 db:77 0.01% | 8 |
| Sensitivity | Kraken2 db:77 non-gut | 8 |
| Sensitivity | Kraken2 db:16 0.01% | 7 |
| Sensitivity | Kraken2 db:16 1000 | 7 |
| Sensitivity | Kraken2 db:16 500 | 7 |
| Sensitivity | Kraken2 db:8 0.01% | 7 |
| Sensitivity | Kraken2 db:16 100 | 7 |
| Sensitivity | Kraken2 db:8 1000 | 7 |
| Sensitivity | Kraken2 db:8 100 | 7 |
| Sensitivity | Kraken2 db:8 500 | 7 |
| Sensitivity | Kraken2 db:8 0.1% | 4 |
| Sensitivity | Kraken2 db:8 gut | 4 |
| Sensitivity | Kraken2 db:16 gut | 4 |
| Sensitivity | Kraken2 db:77 0.1% | 3 |
| Sensitivity | Kraken2 db:16 non-gut | 3 |
| Sensitivity | Kraken2 db:16 0.1% | 3 |
| Sensitivity | Kraken2 db:8 non-gut | 2 |
| True Positive (Abundance of Species Common to Both the Gold Standard and Predicted Groups) | Kraken2 db:77 | 19 |
| True Positive (Abundance of Species Common to Both the Gold Standard and Predicted Groups) | Kraken2 db:77 gut | 13 |
| True Positive (Abundance of Species Common to Both the Gold Standard and Predicted Groups) | Kraken2 db:77 100 | 10 |
| True Positive (Abundance of Species Common to Both the Gold Standard and Predicted Groups) | Kraken2 db:77 500 | 9 |
| True Positive (Abundance of Species Common to Both the Gold Standard and Predicted Groups) | Kraken2 db:16 | 9 |
| True Positive (Abundance of Species Common to Both the Gold Standard and Predicted Groups) | Kraken2 db:8 | 8 |
| True Positive (Abundance of Species Common to Both the Gold Standard and Predicted Groups) | Kraken2 db:77 1000 | 8 |
| True Positive (Abundance of Species Common to Both the Gold Standard and Predicted Groups) | Kraken2 db:77 0.01% | 8 |
| True Positive (Abundance of Species Common to Both the Gold Standard and Predicted Groups) | Kraken2 db:77 non-gut | 8 |
| True Positive (Abundance of Species Common to Both the Gold Standard and Predicted Groups) | Kraken2 db:16 0.01% | 7 |
| True Positive (Abundance of Species Common to Both the Gold Standard and Predicted Groups) | Kraken2 db:16 1000 | 7 |
| True Positive (Abundance of Species Common to Both the Gold Standard and Predicted Groups) | Kraken2 db:16 500 | 7 |
| True Positive (Abundance of Species Common to Both the Gold Standard and Predicted Groups) | Kraken2 db:8 0.01% | 7 |
| True Positive (Abundance of Species Common to Both the Gold Standard and Predicted Groups) | Kraken2 db:16 100 | 7 |
| True Positive (Abundance of Species Common to Both the Gold Standard and Predicted Groups) | Kraken2 db:8 1000 | 7 |
| True Positive (Abundance of Species Common to Both the Gold Standard and Predicted Groups) | Kraken2 db:8 100 | 7 |
| True Positive (Abundance of Species Common to Both the Gold Standard and Predicted Groups) | Kraken2 db:8 500 | 7 |
| True Positive (Abundance of Species Common to Both the Gold Standard and Predicted Groups) | Kraken2 db:8 0.1% | 4 |
| True Positive (Abundance of Species Common to Both the Gold Standard and Predicted Groups) | Kraken2 db:8 gut | 4 |
| True Positive (Abundance of Species Common to Both the Gold Standard and Predicted Groups) | Kraken2 db:16 gut | 4 |
| True Positive (Abundance of Species Common to Both the Gold Standard and Predicted Groups) | Kraken2 db:77 0.1% | 3 |
| True Positive (Abundance of Species Common to Both the Gold Standard and Predicted Groups) | Kraken2 db:16 non-gut | 3 |
| True Positive (Abundance of Species Common to Both the Gold Standard and Predicted Groups) | Kraken2 db:16 0.1% | 3 |
| True Positive (Abundance of Species Common to Both the Gold Standard and Predicted Groups) | Kraken2 db:8 non-gut | 2 |

**Table S7.** Information for the taxonomy classification of experimentally verified and studied enzymes CA-KR1, CA_201 and AL_17, discovered by the seek mode of *ProteoSeeker*. For each enzyme the species of the hit with the lowest E-value, acquired from running the enzyme against the nr protein database through the online suite of blastp, is provided. For the Kraken2 and COMEBin/MetaBinner taxonomy routes, the taxonomy classification of the enzyme is provided. If the taxonomy classification could not be inferred for the protein, then “None” is noted. For the Kraken2 taxonomy route three databases were used for the analysis, the Kraken 2 / Bracken Refseq indexes of the Standard-8 (“Kraken2 db:8”), Standard-16 (“Kraken2 db:16”) and Standard (“Kraken2 db:77”) collections. The COMEBin/MetaBinner taxonomy route was applied based on the non-redundant (nr) protein database of NCBI through COMEBin (“COMEBin db:nr”) and through MetaBinner (“MetaBinner db:nr”). No filtering threshold was applied to the abundances or relative abundances of the taxa predicted by each taxonomy route.

| **Tool and Database** | **CA-KR1** | **CA_201** | **AL_17** |
| --- | --- | --- | --- |
| COMEBin db:nr | *Pyrobaculum aerophilum* | *Novosphingobium*  *Novosphingobium* sp. *AAP1*  *Novosphingobium* sp. *BK256*  *Novosphingobium* sp. *BK280*  *Novosphingobium* sp. *BK258*  *Novosphingobium* sp. *BK267*  *Novosphingobium* *pokkalii* | *Rheinheimera* sp. *MM224* |
| MetaBinner db:nr | None | *Novosphingobium*  *Novosphingobium* sp. *AAP1*  *Novosphingobium* sp. *BK256*  *Novosphingobium* sp. *BK280*  *Novosphingobium* sp. *BK258*  *Novosphingobium* sp. *BK267*  *Novosphingobium* *pokkalii* | *Rheinheimera* sp. *MM224* |
| Kraken2 db:8 | None | *Novosphingobium humi* | *Rheinheimera mangrovi* |
| Kraken2 db:16 | None | *Novosphingobium humi* | *Rheinheimera mangrovi* |
| Kraken2 db:77 | *Moraxella bovoculi* | *Novosphingobium humi* | *Rheinheimera* sp. *MM224* |
| BLASTP db:nr | *Pyrobaculum aerophilum* | *Novosphingobium* sp. | *Rheinheimera mesophila* |

# References

[1] C. Poussin, L. Khachatryan, N. Sierro, V. K. Narsapuram, F. Meyer, V. Kaikala, V. Chawla, U. Muppirala, S. Kumar, V. Belcastro, J. N. D. Battey, E. Scotti, S. Boué, A. C. McHardy, M. C. Peitsch, N. V Ivanov, J. Hoeng, *BMC Genomics* 2022, *23*, 624.

[2] ID 669653 - BioProject - NCBI, https://www.ncbi.nlm.nih.gov/bioproject/?term=PRJNA669653, Accessed on 07/02/2025.

[3] T. Burhanoğlu, Y. Sürmeli, G. Şanlı-Mohamed, *Int J Biol Macromol* **2020**, *164*, 578.
